# The putative polyamine transporter Shp2 facilitates phosphate export in an Xpr1-independent manner and contributes to high phosphate tolerance

**DOI:** 10.1016/j.jbc.2024.108056

**Published:** 2024-12-09

**Authors:** Tochi Komamura, Tomoki Nishimura, Naoki Ohta, Masahiro Takado, Tomohiro Matsumoto, Kojiro Takeda

**Affiliations:** 1 Faculty of Science and Engineering, Department of Biology, Konan University, Kobe, Japan; 2 Radiation Biology Center, Graduate School of Biostudies, Kyoto University, Kyoto, Japan; 3 Institute of Integrative Neurobiology, Konan University, Kobe, Japan

**Keywords:** phosphate, phosphate export, Xpr1, SPX, polyamine, primary familial brain calcification (PFBC), fission yeast, Schizosaccharomyces pombe

## Abstract

Phosphate (Pi) homeostasis at the cellular level is crucial, requiring coordinated Pi uptake, storage, and export. However, the regulatory mechanisms, particularly those governing Pi export, remain elusive, despite their relevance to human diseases like primary familial brain calcification. While Xpr1, conserved across eukaryotes, is the only known Pi exporter, the existence of additional Pi exporting factors is evident; however, these factors have been poorly characterized. Using the fission yeast *Schizosaccharomyces pombe* as a model, we have aimed to better understand cellular Pi homeostasis mechanisms. Previously, we showed three Pi regulators with SPX domains to be critical: Pqr1 (Pi uptake restrictor), Xpr1/Spx2, and the VTC complex (polyphosphate synthase). SPX domains bind to inositol pyrophosphate, modulating Pi regulator functions. The double mutant *Δpqr1Δxpr1* hyper-accumulates Pi and undergoes cell death under high Pi conditions, indicating the necessity of both Pi uptake restriction and export. Notably, *Δpqr1Δxpr1* exhibits residual Pi export activity independent of Xpr1, suggesting the presence of unidentified Pi exporters. To uncover these cryptic Pi exporters and regulators of Pi homeostasis, we conducted suppressor screening for high Pi hypersensitivity in *Δpqr1Δxpr1*. Among the eight suppressors identified, Shp2, a plasma-membrane protein, showed Pi export-facilitating activity in an Xpr1-independent manner, supporting cell proliferation at high Pi. The present results provide the first evidence for Pi export facilitator other than the established Xpr1, unprecedented in eukaryotes. As Shp2 is orthologous to the budding yeast Tpo1, a spermidine/polyamine transporter, a potential link between Pi homeostasis and polyamine metabolism can be speculated.

Phosphate (Pi) homeostasis is essential for cellular function, requiring precise regulation of Pi storage, consumption, import, and export. Studies using the budding yeast *Saccharomyces cerevisiae* have elucidated the PHO pathway, which controls the transcription of genes that maintain Pi homeostasis (1, 2). Despite these advances, the molecular mechanisms of eukaryotic Pi homeostasis at the cellular level, especially in metazoans, remain largely elusive, particularly regarding Pi export, even in yeasts (3, 4, 5, 6).

In eukaryotes, the membrane protein Xpr1 facilitates Pi export. Mammalian XPR1 (xenotropic and polytropic murine leukemia retrovirus receptor) was initially identified as a receptor for viral infection (7, 8, 9), with its Pi-exporting activity reported later (10). XPR1, localized at the plasma membrane, is an eight-transmembrane protein conserved across eukaryotes, possessing an SPX (Syg1-Pho81-Xpr1) domain at its N-terminus and an EXS (Erd1-Xpr1-Syg1) domain in its central part. The SPX domain, found in regulators of Pi metabolism such as Pho81 in the PHO pathway and the VTC complex synthesizing polyphosphate, reportedly functions as a “Pi sensor” by binding to inositol pyrophosphates (PP-IPs) (11). Additionally, PP-IP is reported to bind to the C-terminal region of Pho81, separate from the SPX domain (12). The abundance of PP-IPs fluctuates with cellular Pi concentration, suggesting SPX protein functions are modulated by PP-IPs. While fungi and plants have multiple SPX factors, metazoans possess only Xpr1 (11, 13). In mammals, 1,5-IP_8_ (PP-IP signaling molecule) binding to the SPX domain of XPR1 is necessary for Pi export (14). The importance of XPR1-mediated Pi export is highlighted by its association with primary familial brain calcification, Fanconi syndrome, and serum [Pi] (15, 16, 17).

Xpr1 is the only identified factor facilitating Pi export in eukaryotes; however, it remains controversial whether Xpr1 exclusively mediates Pi efflux in animal cells (18). Xpr1 knockdown or knockout studies demonstrated a significant reduction in Pi efflux but with 25% or more residual Pi efflux activity (9, 10, 18, 19). Regarding the precise molecular mechanisms of Xpr1-dependent Pi export, recent structural analyses of Xpr1 using cryo-EM have suggested the Pi exporting route in the Xpr1 molecule. The opening and closing of this route are regulated by 1,5-IP_8_ binding to the SPX domain (20, 21). In contrast, other recent studies suggested that Xpr1 may be a regulator of cellular Pi homeostasis rather than a Pi exporter (22, 23).

The fission yeast *S. pombe*, like *S. cerevisiae*, serves as a model organism for eukaryotic cell biology. While research on Pi homeostasis in fission yeast has historically lagged behind detailed studies in budding yeast, recent advancements have rapidly improved our understanding of Pi homeostasis in *S. pombe* (6, 24, 25, 26, 27); the SPX domain-containing ubiquitin ligase Pqr1 (similar to the plant *NLA* (28, 29, 30)) is crucial by restricting Pi uptake through the downregulation of high-affinity Pi transporters Pho84 and Pho842 (24). Pqr1/Spx1 is also suggested to be involved in the transcriptional regulation of Pho1 (secreted acid phosphatase) and Pho84 (Pi transporter), both members of the PHO regulon, which are essential for Pi homeostasis (31). Regarding cellular Pi efflux, *S. pombe* possesses a homolog of human XPR1, Xpr1/Spx2, which contributes to cellular Pi homeostasis through its Pi-exporting activity (6). Utilizing *S. pombe*, Pi export dependent on Xpr1 was first demonstrated in fungi. In *S. cerevisiae*, the Pi exporting activity of Syg1, the Xpr1 ortholog, has not been shown, while exocytosis-mediated Pi export was reported (32).

In our previous study, we reported that the normal proliferation of *S. pombe* requires Pi homeostasis maintained by the synergy of three SPX factors (Fig. 1*A*) (6): Pqr1, restricting Pi uptake; Xpr1, an SPX-EXS type Pi export facilitator; and the VTC complex, a polyphosphate synthase with subunits possessing the SPX domain (24, 31, 33). We hypothesized that these three SPX factors prevent lethal increases in cytoplasmic/nuclear [Pi]. During our investigation of Xpr1-dependent Pi export, we realized the possibility that *S. pombe* possesses unidentified Pi exporters in addition to Xpr1. Specifically, a low but significant Pi export activity remains in strains lacking Xpr1. As previously described, the existence of unidentified Pi exporters other than Xpr1 is also predicted in metazoans (18, 22). Given the physiological importance of Pi efflux, particularly in medical contexts, identifying such cryptic Pi exporters is of significant value. In this study, we sought to identify novel Pi exporters and regulators of Pi homeostasis by screening multicopy suppressors of Pi hypersensitivity in the *Δpqr1Δxpr1* double gene deletion mutant.

**Figure 1:**
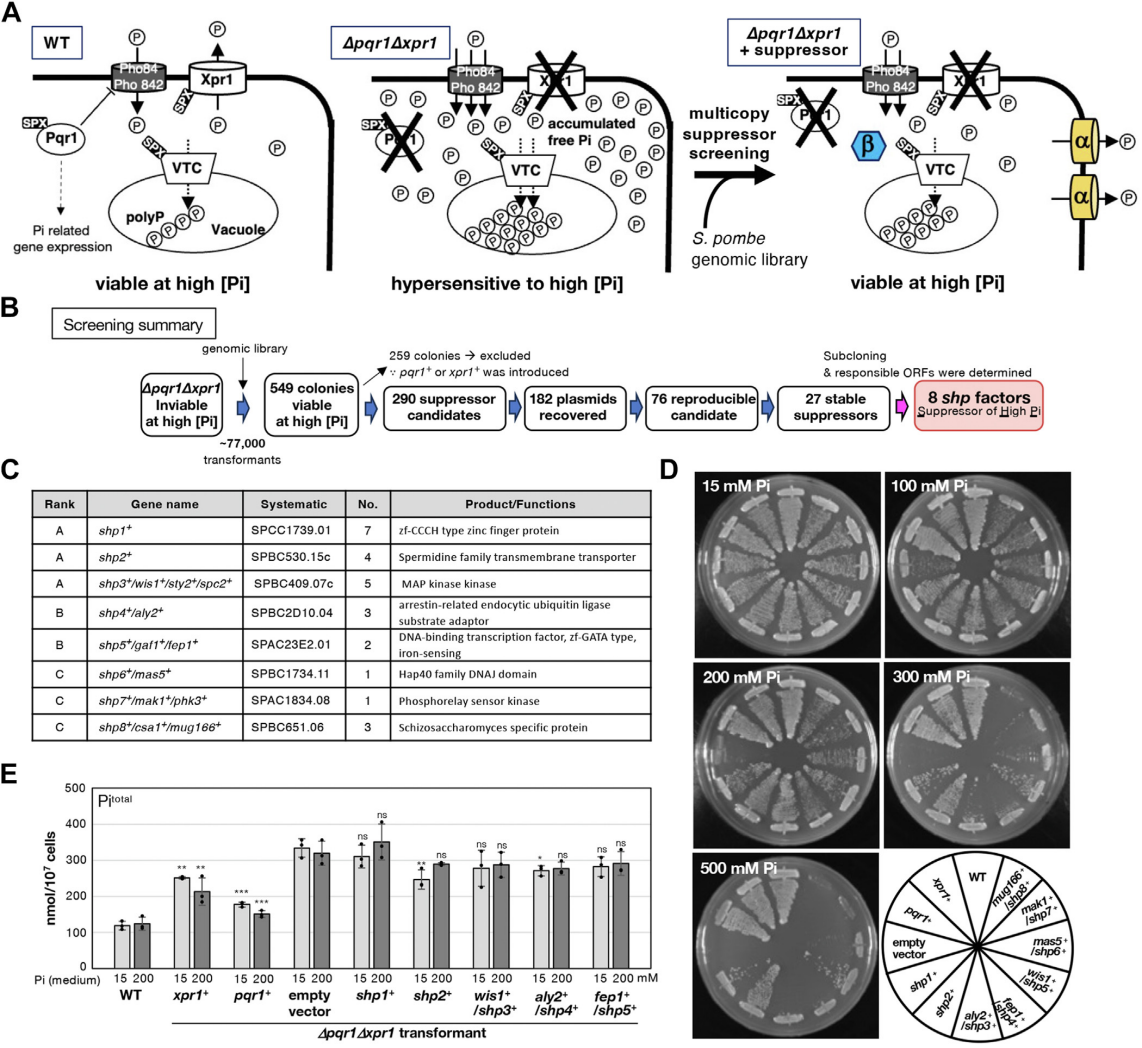
**Eight multicopy suppressors for Pi hypersensitivity of *Δpqr1Δxpr1*.***A*, model for Pi homeostasis in *S. pombe*, regulated by Pqr1, Xpr1, and the VTC complex (*left*). In *Δpqr1Δxpr1*, neither Pi uptake restriction (Pqr1) nor Pi export (Xpr1) is functional, leading to hypersensitivity to high [Pi] (*middle*). Dosage increase of genes of unidentified Pi exporter (α) or Pi regulator (β) may restore Pi homeostasis and support colony-formation at high [Pi] (*right*): the principle of multicopy suppressor screening. *B*, procedure for multicopy suppressor screening using a genomic library. *C*, list of *shp* genes. Rank A: viable at 500 mM Pi. Rank B: viable at 300 mM Pi. Rank C: viable at 200 mM Pi. No. for the number of independent isolates. *D*, *Δpqr1Δxpr1* transformed with plasmids containing *shp* genes restored growth at higher [Pi]. WT indicates wild type 972. Other strains are *Δpqr1Δxpr1* transformants. *E*, total intracellular Pi amounts of indicated strains 8 h after shifting to medium with 15 mM or 200 mM Pi. Data are normalized to cell number. Experiments were repeated 3 × . Individual data points, means, and SDs are presented. One-sided Dunnett test to confirm significant increase of Pi export (empty vs. transformants): ∗∗∗ for *p* < 0.001, ∗∗ for *p* < 0.01, and ∗ for *p* < 0.05.

## Results

### Multicopy suppressors for Pi hypersensitivity of *Δpqr1Δxpr1*

While the WT strain (972*h*^*-*^) proliferates even at 500 mM Pi, colony formation of *Δpqr1Δxpr1* is significantly inhibited at 100 mM Pi, as neither Pi uptake restriction by Pqr1 nor Pi export by Xpr1 is functional (6) (Fig. 1*A*). The standard synthetic medium EMM2 contains Pi at 15 mM. Utilizing the *S. pombe* genomic DNA library pTN-L1 (average insert length = 8 kb, library size = 60,000, (34)), we screened multicopy suppressor genes, dosage increases of which alleviate the Pi hypersensitivity of the *Δpqr1Δxpr1* double gene deletion mutant, aiming to identify novel factors involved in Pi homeostasis, especially unidentified Pi exporters (Fig. 1, *A* and *B* and Supplementary information for the detailed procedure). First, *Δpqr1Δxpr1* was transformed with the genomic library, and we obtained approximately 77,000 colonies, which were subsequently examined for colony formation at higher [Pi] (200 ∼ 500 mM), resulting in 549 transformants viable at [Pi] > 200 mM. Considering the property of pTN-L1 and the number of obtained transformants, the coverage was estimated to be 44, indicating any region of the genome was screened 44 times in average. After excluding colonies containing plasmids with *pqr1*^*+*^ or *xpr1*^*+*^, false-positive candidates, and plasmids with sequencing difficulties (due to plasmids rearrangement, *etc*.) (Fig. 1*B*), we obtained 27 plasmids, stably suppressing the Pi hypersensitivity of *Δpqr1Δxpr1*. The false-positive ratio was ∼48% (the candidates with *pqr1*^*+*^ or *xpr1*^*+*^ were counted as true positives). Then the inserted genomic sequences of the 27 plasmids were analyzed. One of the 27 plasmids contained *pqr1*^*+*^ and the others were classified into eight groups based on the inserted genomic regions (Fig. S1). We determined the responsible ORFs for the suppression and finally identified eight genes as multicopy suppressors for Pi hypersensitivity of *Δpqr1Δxpr*1, designated as *shp1*^*+*^ to *shp8*^*+*^ (Suppressor of High Pi) (Figs. 1, *C* and *D*, S1 and Table S1). As shown in Fig. 1*C*, six of the eight *shp* genes were isolated independently multiple times, indicating the screening was nearly saturated.

The *shp* genes were categorized into three ranks (A to C) based on their suppression strength. Rank A *shp* genes are the most potent suppressors, almost equivalent to *pqr1*^*+*^ or *xpr1*^*+*^, while rank C represents the weakest suppression. Among the eight, *shp2*^*+*^, a rank A suppressor, encodes a transmembrane protein belonging to the major facilitator superfamily (MFS), and none of the eight have so far been reported as regulators for Pi homeostasis.

### Defective Pi homeostasis of *Δpqr1Δxpr1* is partially suppressed by *shp2*^+^*and shp4*^+^

To gain insight into the suppression mechanisms of Pi hypersensitivity, we investigated the impact of increased dosage of *shp1*^*+*^ to *shp5*^*+*^, which belong to rank A or B, on intracellular total Pi amounts (Pi^total^), the sum of free Pi, polyphosphate, and Pi covalently bonded to organic compounds (Fig. 1*E*). WT cells and *Δpqr1Δxpr1* transformants were cultured in EMM2 media containing 15 mM or 200 mM Pi, and their intracellular Pi^total^ were quantified. Consistent with our previous study (6), Pi^total^ in *Δpqr1Δxpr1* cells was threefold higher than in WT cells and was suppressed by either *pqr1*^*+*^ or *xpr1*^*+*^. Transforming *Δpqr1Δxpr1* with plasmids containing *shp2*^*+*^ and *shp4*^*+*^ significantly reduced Pi^total^ compared to *Δpqr1Δxpr1* with an empty vector at 15 mM [Pi]. At 200 mM [Pi], the difference between the empty vector and *shp2*^*+*^ or *shp4*^*+*^ was reproducibly observed but not significant. These results suggest that increased dosage of *shp2*^*+*^ or *shp4*^*+*^ alleviates abnormality in Pi homeostasis of *Δpqr1Δxpr1* to some extent. In contrast, the increase in dosage of *shp1*^*+*^, despite being a strong rank A suppressor, did not reduce Pi^total^ in *Δpqr1Δxpr1* cells.

### Multicopy increase in *shp2*^*+*^, the putative spermidine transporter, restores Pi export activity of *Δpqr1Δxpr1*

**Figure 2:**
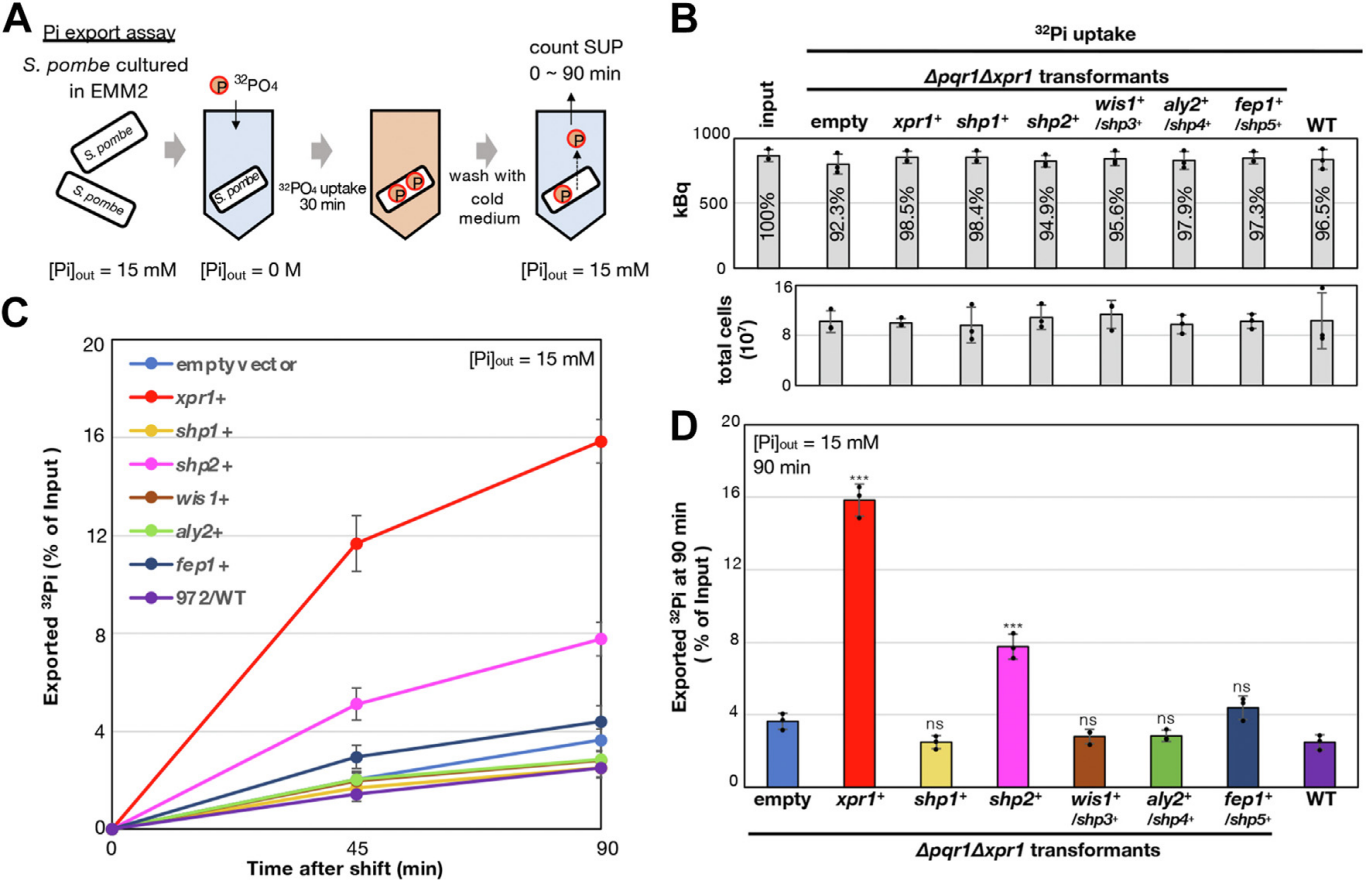
**The putative polyamine/spermidine transporter Shp2 has Pi export activity.***A*, scheme of Pi export assay. *B*, ^32^Pi uptake before the shift. After ^32^Pi incorporation, cell aliquots were analyzed using a liquid scintillation counter. The input and incorporated ^32^Pi amounts are compared (*top*, percentages). Cell numbers used in the assay are also shown (*bottom*). *C*, time-course analysis of ^32^Pi export in 15 mM Pi EMM2. The Y axis shows percentages of ^32^Pi exported relative to total incorporated ^32^Pi. *D*, comparison of exported ^32^Pi at 90 min after the chase. One-sided Dunnett test (empty vs. *shp* transformants) to confirm significant increase of Pi export: ∗∗∗ for *p* < 0.001 and ns for non-significant (*p*≥ 0.05). *B*–*D*, experiments were repeated 3 × . Individual data points, means, and SDs are presented.

To understand the mechanisms underlying *shp* suppression, we investigated the impact of *shp* genes on Pi export activity using ^32^Pi radioisotope (Fig. 2*A*). Previous studies from our lab demonstrated two key findings: firstly, Xpr1 exhibits Pi export activity in *S. pombe*, and secondly, this Xpr1-dependent Pi export is activated in the absence of functional Pqr1 (6). WT cells and *Δpqr1Δxpr1* cells transformed with either an empty vector or plasmids containing *shp* genes were cultured in 15 mM Pi medium, washed, and suspended in Pi-free EMM2-P medium, followed by administration of KH_2_^32^PO_4_. Efficient ^32^Pi incorporation was confirmed in all tested strains (Fig. 2*B*), and their Pi export activities were measured using a liquid scintillation counter (Fig. 2, *C* and *D*). Consistent with our previous study (6), *Δpqr1Δxpr1* transformed with *xpr1*^*+*^ significantly exhibited robust Pi export activity, while those transformed with the empty vector did not, indicating Xpr1-dependent Pi export. In the presence of Pqr1, the primary regulator of Pi homeostasis in *S. pombe*, Xpr1 is rather inactive, resulting in relatively low Pi export activity in WT cells, as previously reported. Surprisingly, *Δpqr1Δxpr1* transformed with *shp2*^*+*^ multicopy plasmid displayed strong Pi export activity equivalent to 50% of that of *xpr1*^*+*^. The difference between the empty vector and *shp2*^*+*^ was significant. Notably, as the *xpr1*^*+*^ gene is deleted in *Δpqr1Δxpr1*, the upregulation of *shp2*^*+*^ dosage promoted Pi export in an Xpr1-independent manner. Conversely, *shp1*^*+*^ and *shp3*^*+*^ to *shp5*^*+*^ did not promote Pi export (Fig. 2, *C* and *D*). These findings suggest that Shp2 suppresses Pi hypersensitivity in *Δpqr1Δxpr1* by enhancing Pi export activity independent of Xpr1. The primary objective of our study is to identify the cryptic Pi exporter predicted in our previous research. Shp2, a putative spermidine (SPD) transporter with a twelve-transmembrane domain, emerged as a promising candidate for this cryptic unidentified Pi transporter.

### Shp factors other than Shp2, a putative SPD transporter

Although Shp1 and 3 ∼ 8 were not investigated further in the present study because our primary goal was to discover unidentified Pi exporters, all seven will be pivotal in cellular Pi homeostasis and, therefore, should be investigated in the future. Here we provide some information on the seven Shp factors.

Shp1 and Shp3 are strong rank A suppressors other than Shp2. Shp1 is a poorly characterized protein with a CCCH-type zinc finger, potentially related to human Makorin (35), a ubiquitin ligase, and potentially an RNA-binding protein, although the RING finger motif is not conserved in Shp1. The suppression activity of Shp1 is second only to that of Shp2; however, Pi^total^ was not affected by the dosage increase of Shp1 (Fig. 1*C*). Shp3 is Wis1, the MAPK kinase, which activates the stress MAPK Spc1/Sty1 (36, 37). Despite the difference being non-significant (*p* = 0.051), Pi^total^ was reproducibly reduced by Shp3/Wis1 increase (Fig. 1*E*), suggesting that this well-known stress response system could contribute to Pi homeostasis.

Two rank B suppressors are Shp4/Aly2 and Shp5/Gaf1/Fep1. The dosage increase of Shp4/Aly2, a ubiquitin ligase adaptor arrestin, significantly reduced Pi^total^, suggesting its potential involvement in Pi import or export. As the Pi export assay (Fig. 2) showed that Shp4/Aly2 does not enhance Pi export activity in *Δpqr1Δxpr1*, we consider that Shp4/Aly2 may regulate Pi import. The arrestin-like factors promote the endocytosis of transporters in the plasma membrane (38, 39, 40). Of note, the major Pi importer Pho84 is ubiquitinated and down-regulated through endocytosis in a Pqr1-dependent manner, resulting in Pi uptake restriction (24). Shp5 is a GATA factor with two zinc fingers, originally identified as Gaf2; lately, it was identified as Fep1, regulating cellular iron metabolism (41, 42, 43). Shp5 did not significantly affect Pi^total^.

The remaining three rank C suppressors, namely Shp6/Mas5, Shp7/Mak1/Phk3, and Shp8/Csa1/Mug166, were relatively weak but stable suppressors for the Pi hypersensitivity (Fig. 1, *C* and *D*). Shp6/Mas5 is a nucleocytoplasmic type-I HSP40 involved in protein quality control and heterochromatin formation (44, 45). Shp7/Mak1/Phk3 is a histidine kinase, a part of the His-Asp phosphorelay system, responding to environmental stresses (46). Similar histidine kinases, Mak2/Phk1 and Mak3/Phk2, are located upstream of the Wis1-Spc1 stress MAPK pathway (47). Shp8/Csa1/Mug166 is a *Shizosaccharomyces*-specific protein with unknown function, which is transcriptionally activated in meiosis (48).

### Shp2: a putative spermidine/polyamine transporter suppressing high Pi hypersensitivity of Δpqr1Δxpr1

**Figure 3:**
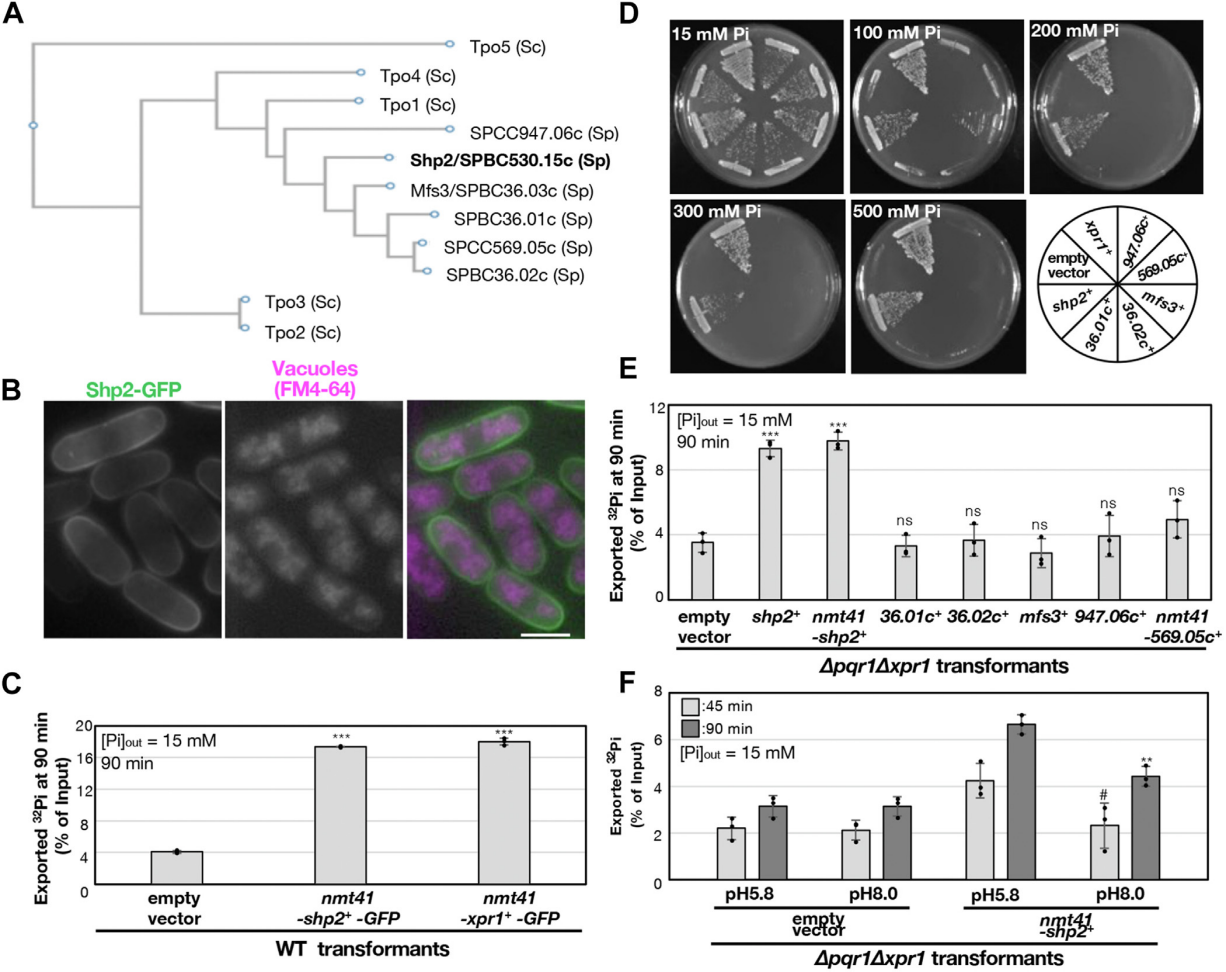
**Shp2, but not other spermidine transporters, possesses Pi exporting activity.***A*, phylogenetic tree of spermidine transporters in *S. pombe* (Sp) and *S. cerevisiae* (Sc). Alignment in Fig. S2. *B*, Shp2-GFP (*green*) localizes to the plasma membrane, not the vacuole membrane (FM4-64, magenta). Bar, 5 μm. *C*, the overproduction of Shp2 led to a four-fold increase in Pi export. *D*, plate assay of *Δpqr1Δxpr1* transformed with multicopy plasmids containing indicated spermidine transporters. 36.01c, 36.02c, 569.05c, and 947.06c represent SPBC36.01c, SPBC36.02c, SPBC569.05c, and SPCC947.06c, respectively. SPCC569.05c is under *nmt41* promoter; others under their own promoters. *E*, ^32^Pi export assay using *Δpqr1Δxpr1* cells transformed with multicopy plasmids expressing indicated spermidine transporters. Experiments were repeated 3 × . Individual data points, means, and SDs are presented. *F*, Shp2-dependent Pi export is pH sensitive. Statistical analyses for *C* and *E*, one-sided Dunnett test to confirm significant increase of Pi export (empty vs. transformants): ∗∗∗ for *p* < 0.001 and ns for non-significant (*p*≥ 0.05). For *F*, two-sided Welch’s *t* test was applied (pH 5.8 vs. pH 8.0). ∗∗ for *p* < 0.01 and # for *p* = 0.051.

The precedent studies using *S. cerevisiae* revealed that Tpo1∼4 are engaged in cellular polyamine export: Tpo1 and four for SPD and spermine (SPM); Tpo2 and three for SPM (49, 50, 51). *S. pombe* harbors six membrane proteins, including Shp2, orthologous to Tpo1 (Fig. 3*A*), although the polyamine transport activity of these six proteins remains unconfirmed. Mfs3, one of the six, was reported to affect drug sensitivity (52). As Tpo1 has been suggested to function at either the vacuolar membrane or the plasma membrane (49, 51, 53), we investigated the cellular localization of Shp2-GFP (Fig. 3*B*). This observation indicates that Shp2-GFP is predominantly localized to the cell periphery, likely the plasma membrane, suggesting that Shp2 primarily functions in the plasma membrane. The Shp2-GFP suppressed the Pi hypersensitivity of *Δpqr1Δxpr1* (Fig. S3).

To confirm the Pi export promoting activity of Shp2, we examined whether the overproduction (OP) of Shp2 accelerates Pi export (Figs. 3*C* and S4). In the previous study, it was demonstrated that Xpr1-OP led to a sixfold increase in Pi export (6). Shp2-GFP or Xpr1-GFP was overproduced with the inducible *nmt41* promoter for 20 h in WT, and the Pi exporting activity was measured. Similar to Xpr1-OP, Shp2-OP led to a fourfold increase in Pi export.

Subsequently, we assessed whether the other five putative SPD transporters exhibit multicopy suppression activity similar to Shp2 in *Δpqr1Δxpr1* (Fig. 3*D*). The five putative SPD transporters reportedly show the cell peripheral localization, suggesting that they function in the plasma membrane (54). However, none of the multicopy plasmids with the five SPD transporters other than Shp2 were able to rescue the growth of *Δpqr1Δxpr1* under elevated Pi conditions. Furthermore, the Pi export assay revealed that the increased dosage of the five did not restore Pi export activity as observed with Shp2 (Figs. 3*E* and S5).

The present results led us to speculate a potential link between Pi and SPD/polyamine metabolisms, as Pi export is facilitated by Shp2, sequentially orthologous to Tpo1, the established SPD/polyamine transporter. To gain more insight, we first examined whether Tpo1 expression suppresses the Pi hypersensitivity of *Δpqr1Δxpr1*. Tpo1 was expressed from the multicopy plasmids with the inducible promoter *nmt41* in *Δpqr1Δxpr1* on EMM2 with various Pi concentrations (Fig. S6). The colony formation of *Δpqr1Δxpr1* was reproducibly confirmed at higher Pi when Tpo1 was overproduced. Second, we examined whether Shp2-dependent Pi export is affected by pH (Figs. 3*F* and S7), as the SPD exporting activity of Tpo1 is pH sensitive: more active at lower pH. Shp2 was overproduced in *Δpqr1Δxpr1* and the Pi export assay was performed at pH 5.8 and 8.0. In Shp2-OP, the Pi exporting activity was significantly higher at pH 5.8 than at pH 8.0, suggesting the Shp2-dependent Pi export may be active in acidic conditions, as observed in SPD export by Tpo1. These results suggest Shp2 may be a functional ortholog of Tpo1, although its SPD exporting activity is not examined yet.

Together, we conclude the putative SPD transporter Shp2 facilitates the Xpr1-independent Pi export activity in *S. pombe*.

### Endogenous Shp2 contributes to Pi tolerance and export at elevated Pi concentrations

**Figure 4:**
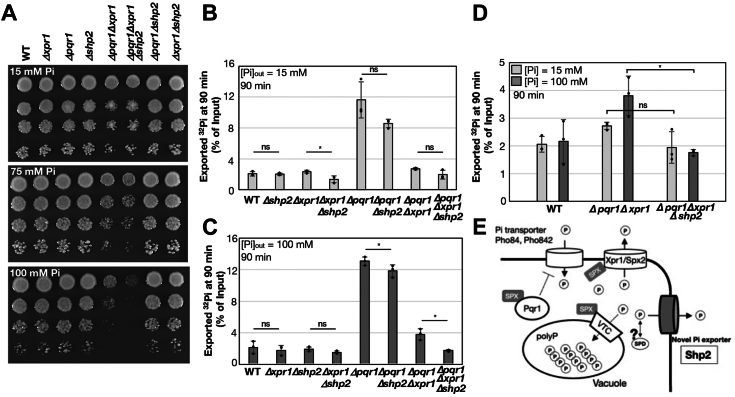
**The gene deletion of *shp2***^**+**^**reduces Pi export activity.***A*, spot assay at higher [Pi]. *Δpqr1Δxpr1Δshp2* exhibits more severe Pi hypersensitivity. *B*–*D*, ^32^Pi export assay using indicated strains at 90 min after the chase. [Pi] in the medium was 15 mM (*B*) or 100 mM (*C*). *D*, compares ^32^Pi export activities of WT, *Δpqr1Δxpr1*, and *Δpqr1Δxpr1Δshp2* at 100 mM Pi to those at 15 mM Pi, which are shown in Fig. 4, *B* and *C*. Experiments repeated 3 × . Individual data points, means, and SDs presented. One-tailed Welch’s *t* test to confirm significant reduction of Pi export by *shp2*^+^ deletion (*shp2*^+^ vs. *Δshp2*): ∗ for 0.01 < *p* < 0.05 and ns for non-significant (*p*≥ 0.05). E. Summary: Shp2, a putative spermidine transporter, independently promotes Pi efflux alongside established Pi exporter Xpr1. SPD denotes spermidine.

To assess the involvement of Shp2 in Pi homeostasis under more physiological conditions, we examined Pi hypersensitivity in gene deletion mutants of Shp2, Pqr1, and Xpr1 (Fig. 4*A*). While *Δpqr1Δxpr1* displayed limited growth at 100 mM Pi as also shown in Figure 1, *Δpqr1Δxpr1Δshp2* exhibited reproducibly more severe growth defect than *Δpqr1Δxpr1* at 100 mM Pi, indicating the importance of Shp2 in cellular proliferation in the absence of Xpr1 and Pqr1. These results suggest Xpr1 and Shp2 synergistically contribute to high Pi tolerance.

Subsequently, Pi export assays were performed on these gene deletion mutants (Figs. 4*B* and S8). Initially, Pi export activities were quantified in normal EMM2 medium with 15 mM Pi. The Pi export activity of *Δpqr1* was higher than that of WT, consistent with our previous study (6). We analyzed whether *shp2*^*+*^ deletion reduced Pi export in different genetic backgrounds shown in Fig. 4*B*. *Δxpr1Δshp2* exhibited significantly lower Pi export activity than *Δxpr1*. Pi exports of *Δpqr1Δshp2* were reproducibly lower than *Δpqr*1, although the difference was not statistically significant (*p* = 0.079). Shp2-dependent Pi export likely exists, although its activity may be rather minor at 15 mM Pi.

Given the potential requirement of Shp2 in the absence of both Pqr1 and Xpr1 at higher Pi concentrations, Pi export activities were examined at 100 mM Pi (Figs. 4, *C* and *D*, S8). This time, *shp2*^*+*^ deletion significantly reduced Pi exporting activity: *Δpqr1* vs. *Δpqr1Δshp2*; *Δpqr1Δxpr1* vs. *Δpqr1Δxpr1Δshp2*. This result underscores the existence of Shp2-dependent Pi export.

In summary, our findings suggest that the putative polyamine exporter Shp2, identified in our genetic screening, functions as a second Pi export facilitator on the plasma membrane and synergizes with Xpr1 to support cellular proliferation at elevated Pi concentrations.

## Discussion

In this study, we aimed to identify unidentified Pi exporters and novel regulators for Pi homeostasis in *S. pombe*. We conducted genetic screening of multicopy suppressors for high Pi hypersensitivity of *Δpqr1Δxpr1*, isolating eight multicopy suppressor genes, designated *shp1*^*+*^∼*8*^*+*^. Among them, the membrane protein Shp2 was found to possess Pi export facilitating activity, suggesting its role as the unidentified Xpr1-independent Pi exporter predicted in previous studies (6, 18, 22). Shp2 represents the first Pi export facilitator on the plasma membrane other than Xpr1. The Shp2-dependent Pi export likely contributes to Pi homeostasis, as evidenced by the synergistic support for growth with Xpr1 at higher Pi concentrations.

Shp2, a 12-transmembrane protein, belongs to the major facilitator superfamily (MFS). *S. cerevisiae* counterparts of Shp2 are Tpo1 and Tpo4, transporters of polyamines (SPD and SPM), which export or import polyamines at acidic or alkaline pH (49, 50, 51). Shp2 and Tpo1/4 share homology with *S. cerevisiae* Tpo2 and Tpo3, SPM transporter (50). Tpo1∼4 belong to the DHA1 family transporter with broad substrate specificities, conferring multi-drug resistance (55). Tpo2 and Tpo3 are involved in the efflux of acetate, a typical anion, although Pi exporting activities of Top1∼4 have not been reported yet. *S. pombe* possesses six putative SPD exporters including Shp2 (Fig. 3*A*), which are 36 ∼ 54% identical to each other (Fig. S2). Among the six, only Shp2 has a detectable Pi export-promoting activity and suppresses the Pi hypersensitivity of *Δpqr1Δxpr1*. Like Tpo2/3, Pi export by Shp2 could reflect its broader substrate recognition capability. Interestingly, Mfs3, one of the six, is transcriptionally upregulated upon either increasing IP_8_, a key signaling molecule in Pi homeostasis, or decreasing medium [Pi], implying a potential link between Mfs3 and Pi regulation (25, 56). Hence, we do not exclude the possibility that the five SPD transporters other than Shp2 may contribute to Pi homeostasis to some minor extent. It is worth examining whether the over-production of the five with the strong *nmt1* promoter rescues *Δpqr1Δxpr1* or facilitates Pi export. *Δxpr1Δshp2* still showed residual Pi exporting activity (Fig. 4), suggesting minor Pi exporter(s) exist. Further research is warranted to elucidate the domains or residues of Shp2 responsible for Pi export by comparing Shp2 and other related transporters.

Currently, proteins highly similar to Shp2 in mammals have not been identified. Shp2 is a member of the MFS comprising ∼105 families. The Pi transporters in mammals, such as type I (NPT, SLC17 family), type II (SLC34 family), and type III (SLC20 family) Na^+^/Pi cotransporters (3), belong also to the MFS. Future research aimed at identifying human Pi exporters other than Xpr1, through scrutiny of amino acid sequences and also the structures of these transporters, is imperative.

This study suggests that the two distinct Pi exporting pathways, namely Xpr1-dependent and Shp2-dependent pathways, synergistically achieve high Pi tolerance. Pi export assays using gene deletion mutants demonstrated the Pi export facilitating activity of endogenous Shp2. Notably, the increased Pi export of *Δpqr1Δxpr1* with higher extracellular [Pi] is nullified upon Shp2 deletion, indicating a regulatory response to extracellular Pi fluctuation. Shp2 likely reinforces Pi export that mainly depends on Xpr1, particularly at elevated external [Pi], synergistically maintaining cellular viability through Pi homeostasis. While recent two excellent studies solved the Xpr1 structure and proposed its unique mechanisms of Pi export (20, 21), cellular Pi export remains enigmatic. For example, the energy dependency of Xpr1 is unclear in the presented models. Our study demonstrated that Xpr1 contributes to Pi homeostasis at very high [Pi], over 200 mM, suggesting that Xpr1 may export Pi against the steep Pi concentration gradient. As for Shp2, potentially a member of the DHA1 family, the mechanism will be based on proton co-transportation, a typical type of active transportation. Other recent studies regarding Xpr1 provided contrasting viewpoints: Xpr1 is involved in Pi homeostasis by regulating the stability of Pi importer PiT-1 (23); Xpr1 is not a direct Pi exporter and controls other Pi regulators (22). In our present study, Pi export assay using deletion mutants suggests that Xpr1-dependent Pi export and Shp2-dependent Pi export are distinct. In addition, our previous study demonstrated that Xpr1 overproduction leads to a sixfold increase in Pi export activity, compared to the WT (6). Therefore, Pi may be exported directly through Xpr1, although we do not rule out the possibility that Xpr1 additionally has regulatory roles for other Pi-regulating factors. Now the involvement of Shp2 and potentially SPD/polyamine in Pi export has been revealed in our study and therefore this molecule will be one of the keys to elucidating overall mechanisms of Pi export in eukaryotic cells.

What is the molecular mechanism by which Shp2 facilitates Pi export? The possible mechanisms are broadly categorized into the following two: Pi is transported across the membrane directly through the Shp2 molecule.; Shp2 activates Pi export pathway(s) depending on other factors. In the present study, no definitive evidence was obtained. The direct experimental demonstration requires analyses using the *in vitro* reconstitution system of Shp2-dependent Pi export. We suspect that Shp2 may mediate Pi export directly based on several data obtained in this study: Shp2-dependent Pi export is distinct from Xpr1, as Shp2 promotes Pi export in *Δxpr1*.; Shp2-OP enhances Pi export to a considerable extent.; Shp2-dependent Pi export is pH sensitive as Tpo1-dependent SPD export is (53). It will be crucial to investigate the biochemical properties of Shp2 (*e.g.*, energy dependency, proton gradient dependency).

Is the Pi export facilitating activity of Shp2 dependent on its SPD/polyamine transporting activity? We have no direct and definitive evidence to address the question yet. However, Shp2 activity may be close to Tpo1: first, the increase in dosage of Tpo1 partially rescues *Δpqr1Δxpr1*; second, the Pi export activity of Shp2 is affected by pH, as the SPD export activity of Tpo1 (53). To consolidate the hypothesis that Shp2 is functionally orthologous to Tpo1, the following additional analyses would be useful: examining whether Shp2 expression in *S. cerevisiae tpo1Δ* functionally complements its sensitivity to SPD or H_2_O_2_ (51, 57). SPD is essential for cell proliferation and is implicated in medically significant phenomena like oxidative stress resistance, autophagy, aging, and immune response (57, 58, 59, 60). The potential relevance of polyamines to cellular Pi homeostasis, both fundamental for human health, warrants further investigation. One recent study using *S. cerevisiae* reported the synthesis of polyamines is affected by Pi starvation (61). As described, polyamine transporter Tpo1 confers resistance to broad spectrum of chemicals; however, it is unclear whether the resistance is attributed directly to polyamine transport or not. Of note, Tpo1-mediated SPD and SPM exports are important for oxidative stress responses (57). Electrostatic interaction between Pi and SPD could play a role in Pi homeostasis, considering the cationic nature of polyamines and the anionic nature of Pi. It could be speculated that excess Pi transiently interacts with SPD, and such interaction may promote Pi export *via* SPD exporting mechanisms. This may work as an urgent backup system for the Xpr1-dependent Pi export when the cytoplasmic [Pi] elevates to the lethal level. As we can not exclude the possibility that Shp2 is simply a new Pi exporter not dependent on SPD at present, further investigations are needed to clarify the involvement of SPD/polyamine in Pi export and Pi metabolisms.

We identified seven other *shp* factors, none of which have been reported as Pi regulators. In this report, we focused on investigating Shp2, because our initial goal is the identification of unidentified Pi exporters or facilitators predicted in our previous study (6). Further efforts to understand how these *shp* factors suppress Pi hypersensitivity will be valuable for elucidating the still enigmatic mechanisms of Pi homeostasis at the cellular level in eukaryotes. Our *S. pombe* system enabled us to evaluate Pi export activities with colony assay without ^32^Pi, opening an avenue to full-scale genetic screening as we performed. Hence, this system will apply to identify metazoan or plant factors related to Pi homeostasis including unidentified Pi exporters: multicopy suppressor screening with human cDNA expression libraries.

## Experimental procedures

### Yeast strains, media, and culture

All *S. pombe* strains used are listed in Table S2. Complete and synthetic minimal media, YES (YE with five supplements: adenine, uracil, leucine, histidine, and lysine), and EMM2 were adopted (62). For Pi-increased EMM2 (75–500 mM Pi), the appropriate volume of Pi buffer (pH 5.8, same as EMM2) was added to EMM2. Yeast strains were incubated on agar plates or in liquid media at 26 °C.

### Multicopy suppressor screening

For screening multicopy suppressors, we utilized the *S. pombe* genomic library pTN-L1 (34) provided by the NBRP Japan. Detailed information is provided in the supplementary section.

### Fluorescent microscopy

Cell images were acquired using an Axiovert 200M ﬂuorescence microscope (Carl Zeiss). FM4-64 (ThermoFischer/Invitrogen) was used to stain the vacuolar membrane (63).

### Quantification of cellular total Pi and Pi export assay

To quantify intracellular Pi^total^ and Pi export activity, we followed the procedure described previously (6, 24). Detailed information is provided in the supplementary section.

## Data availability

All the data described are located in this article.

## Supporting information

This article contains supporting information (64).

## Conflict of interest

The authors declare that they have no conflicts of interest with the contents of this article.
