# Supplementary material for: The putative polyamine transporter Shp2 facilitates phosphate export in an Xpr1-independent manner and contributes to high phosphate tolerance
Source: J Biol Chem. 2024 Dec 9;301(1):108056. doi: 10.1016/j.jbc.2024.108056 (PMC11742622; doi:10.1016/j.jbc.2024.108056)
Supplement: Supporting information [file mmc1.docx]

**Supportive Information**

**The putative polyamine transporter Shp2 facilitates phosphate export
in an Xpr1-independent manner and contributes to high phosphate tolerance**

Tochi Komamura^1^, Tomoki Nishimura^1^, Naoki Ohta^1^, Masahiro Takado^3^,
Tomohiro Matsumoto^3^ and Kojiro Takeda^1,2,#^

^1^Department of Biology, Faculty of Science and Engineering,
^2^Institute of Integrative Neurobiology, Konan University, Kobe, Japan

^3^Radiation Biology Center, Graduate School of Biostudies, Kyoto University, Kyoto, Japan

**The List of Supportive Information:**

Experimental procedures (additional)

Table S1. The detailed list of the eight *shp* genes
Table S2. The list of strains used in this study

Figure S1. The procedure of multicopy suppressor screening

Figure S2. The multiple alignments of polyamine transporters in *S. pombe*

Figure S3. Shp2-GFP suppresses the Pi hypersensitivity of *∆pqr1∆xpr1*
Figure S4. Shp2 overproduction accelerates Pi export

Figure S5. Pi export assay of Shp2 and five other polyamine transporters

Figure S6. Tpo1 overproduction rescues *∆pqr1∆xpr1*

Figure S7. Pi export assay at pH 5.8 and 8.0

Figure S8. Pi export of the deletion mutants

**Experimental Procedures (additional)**

**Quantification of cellular total Pi**

To quantify intracellular Pi^total^, we followed the procedure described previously (21). In short, harvested cells were suspended in 1 M H_2_SO_4_ and were boiled at 95 °C for 30 min to hydrolyze and extract intracellular Pi. Then the extract was neutralized with 1N NaOH. [Pi] was measured with a Malachite Green Phosphate Assay Kit (R&DSystems, Inc.).

**Pi export assay**

To quantify Pi exporting activity, we followed the procedure described previously (6). In short, radioactive monopotassium phosphate (KH_2_^32^PO_4_, 74 MBq/mL, NEX060, PerkinElmer) was added to cell suspension in EMM2-P and incubated at 26 °C for 30 min for ^32^Pi uptake. Then, cells were washed and suspended in EMM2 with 15 or 100 mM Pi (Fig. 2, Fig. 3C and E, and Fig. 4). For the pH experiment, cells were suspended in the following buffers:

pH 8.0 buffer: 10 mM Tris-HCl (pH 8.0), 111 mM glucose, 99.5 mM NH_4_Cl, 15.5 mM Na_2_HPO_4_pH 5.8 buffer: 10 mM MES-NaOH (pH 5.8), 111 mM glucose, 99.5 mM NH_4_Cl, 15.5 mM Na_2_HPO_4_

The compositions of these buffers were based on the minimal medium EMM2. As the rise in pH of the EMM2 resulted in the formation of insoluble precipitates, we removed minerals and vitamins from the EMM2 and adjusted the pH as described above.

At the indicated time point, an aliquot of the supernatant was added to a scintillator, Ecoscint^TM^ Ultra (National Diagnostics). Radioactivity was measured with a liquid scintillation counter AccuFLEX LSC-8000 (Hitachi, Japan).

**Multicopy suppressor screening**

To perform multicopy suppressor screening, the double deletion mutant *∆pqr1∆xpr1* (h- *leu1-32* *∆pqr1*::hph *∆xpr1*::kan) was transformed with the *S. pombe* genomic library pTN-L1 (NBRP yeast, ref Nakamura et al. 2001 MBC). To generate the library pTN-L1, the genome DNA of *S. pombe* (~14.1 Mb) was digested with the restriction enzyme Sau3A I (average insert size: 8 kb) and the resulting fragments were cloned into the multicopy vector plasmid pAL-KS (BamHI site, Tanaka et al., 2000, MCB). The library size is approximately 60,000. The probability that the whole regions of the genome are covered in the library is calculated from the equation #1,

$$P_{1}=1-(1-\frac{l}{L})^{n} (\#1)$$

where *P*_1_ is the probability, *L* is the genome size, *l* is the average insert size in a single clone, and *n* is the number of clones in the library (Clarke and Carbon 1976). In this case, *P*_1_ is estimated as ~1, indicating that most parts of the genome were covered in the library. The double mutant *∆pqr1∆xpr1* was transformed with pTN-L1, resulting in ~77,000 colonies. The coverage of the genome in this screening is calculated from the equation #2,

$$\lambda=N\times\frac{l}{L} (\#2)$$

where *N* is the number of colonies obtained from the transformation of *∆pqr1∆xpr1*. In this case, *λ* is estimated as ~43.7, indicating that any region in the genome was covered for ~43.7 times in average.

The 77,000 transformants were subsequently examined for colony formation at higher [Pi] (200 ~ 500 mM). In total, we obtained 549 clones growing on higher Pi plates. The 549 transformants were analyzed with colony PCR amplifying the ORF of *pqr1^+^* or *xpr1^+^* to exclude the transformants harboring plasmid of *pqr1^+^* or *xpr1^+^* because our targets are multicopy suppressors. As a result, 259 strains harbored *pqr1^+^* or *xpr1^+^* and therefore were excluded from further analysis. From the remaining 290 candidates, we succeeded in recovering 182 plasmids, which were introduced again to *∆pqr1∆xpr1* to test the reproducibility of the suppression. Including doubtful (unstable) candidates, 76 plasmids possibly suppressed the Pi hypersensitivity of *∆pqr1∆xpr1*, and were analyzed with conventional Sanger sequencing (Eurofin genomics). The sequence primers are M13 reverse and M13 forward, which flank the genomic insertion. Among the 76 candidates, we selected 27 plasmids as stable multicopy suppressors for the Pi hypersensitivity of *∆pqr1∆xpr1*. The remaining candidates either showed very weak or unstable suppression or were difficult to determine the inserted sequences due to plasmid fusion or other unknown reasons. We focused on analyzing the 27 candidates. As for unreadable plasmids, we will analyze some of them with whole plasmid sequencing.

The inserted genomic regions in the 27 suppressors were classified into nine groups, eight of which are shown in Fig. S1, and were expected to contain a multicopy suppressor gene. The remaining group, not shown in Fig. S1, contained *pqr1^+^* ORF and was therefore excluded from further analysis. The numbers of isolates and their genomic regions are also shown in Fig. S1 (regions 1 to 8). In short, regions 1, 2, and 5, which showed strong suppression activities (~ 500 mM Pi), were independently isolated seven, four, and five times, respectively. The regions 3 and 4 with medium suppression strength (~300 mM Pi) were isolated three and twice. The regions 6 to 8 with weak suppression strength (~200 mM Pi) were isolated once, once, and three times, respectively. The strong suppressors tended to be isolated multiple times. Judging from these results, the library size, and the estimates of the coverage in the screening, the present screening scale is virtually enough and close to saturation, although we do not exclude the possibility of some unidentified suppressors.

To determine the responsible gene for multicopy suppression, we subcloned each ORF with 1,000 bp flanking sequences to pAL vector using high fidelity PCR enzyme (PrimeStar MAX, TAKARA, Japan) and In-Fusion technique (Clontech/TAKARA, Japan). Then, we examined which ORF rescues *∆pqr1∆xpr1* growth at higher [Pi]. Summing up, we concluded that SPCC1739.01, SPBC530.15c, SPBPC2D10.04, SPAC23E2.01, SPBC409.07c, SPBC1734.11, SPAC1834.08 and SPBC651.06 are responsible for the multicopy suppression activities of the region 1 to 8, respectively (Fig. S1). We designated these eight genes *shp1^+^* to *8^+^* (Suppressor of High Pi), summarized in Fig. 1D.

**Table S1. The detailed list of the eight *shp* genes**

|  | **Systematic name** | **Synonym** | **No. of**  **isolates** | ***S. cerevisiae*** | ***H. sapiens*** | **Product/Functions^*^** | **Suppression Rank** |
| --- | --- | --- | --- | --- | --- | --- | --- |
| *shp1^+^* | SPCC1739.01 | - | 7 | LEE1/YPL054W | MKRN1  /HGNC7112 | zf-CCCH type zinc finger protein | A |
| *shp2^+^* | SPBC530.15c | - | 4 | TPO1/YLL028W | - | Spermidine family transmembrane transporter | A |
| *Shp3^+^* | SPBC409.07c | *spc2+, smf2, sty2^+^, wis1^+^* | 5 | PBS2/YJL128c | MAP2K1  MAP2K2 | MAP kinase kinase | A |
| *Shp4^+^* | SPBC2D10.04 | *aly2^+^* | 3 | ALY2/YJL084C  ALY1/YKR021W | - | arrestin-related endocytic ubiquitin ligase substrate adaptor Aly2 | B |
| *Shp5^+^* | SPAC23E2.01 | *gaf2^+^, fep1^+^* | 2 | GZF3/YJL110C  DAL80/YKR034W | - | DNA-binding transcription factor, zf-GATA type, iron-sensing | B |
| *shp6^+^* | SPBC1734.11 | *mas5^+^* | 1 | YDJ1/YNL064C | DNAJA2  DNAJA4  DNAJA4 | Hap40 family DNAJ domain protein | C |
| *shp7^+^* | SPAC1834.08 | *phk3^+^, mak1^+^* | 1 | SLN1/YIL147C | - | Phosphorelay sensor kinase | C |
| *shp8^+^* | SPBC651.06 | *csa1^+^, mug166^+^* | 3 | - | - | Schizosaccharomyces specific protein Mug166 | C |

-: not found, *: quoted from Pombase (<https://www.pombase.org>)

**Table S2. The list of *S. pombe* strains used in this study**

| **Strain** | **Genotype** | **Source** |
| --- | --- | --- |
| 972 | WT h^-^ |  |
| KP573 | h^-^ *∆pqr1*::hphMX | Sawada et al.^21^ |
| TKN46 | h^-^ *∆xpr1*::kanMX | Takado et al.^6^ |
| TKN55 | h^-^ *∆pqr1*::hphMX *∆xpr1*::kanMX | Takado et al.^6^ |
| KmT104 | h^-^ *∆shp2*::bsd | BIONEER derived# |
| KmT128 | h^-^ *∆pqr1*::hphMX *∆xpr1*::kanMX *∆shp2*::bsd | This study |
| KmT135 | h^-^ *∆pqr1*::hphMX *∆shp2*::bsd | This study |
| KmT131 | h^-^ *∆xpr1*::kanMX *∆shp2*::bsd | This study |
| KmT324 | h^-^ *leu1-32* *∆pqr1*::hphMX *∆xpr1*::kanMX | This study |
| KP3 | h^-^ *leu1-32* |  |

#: The original drug marker gene, kanMX, was substituted with bsd, blasticidin S resistant marker gene.

**Figure S1. The procedure of multicopy suppressor screening**

The following figures, Fig. S1A to H, describe the genomic regions inserted into the confirmed multicopy suppressor plasmids and the procedure to determine the responsible ORF for suppressing the Pi hypersensitivity of *∆pqr1∆xpr1*. In short, the possible ORFs were subcloned to pAL multicopy vector with UTRs and flanking regions (1,000 bp for the upstream and 500 bp for the downstream). The resulting plasmids were examined for their suppression abilities. In addition, as for Shp2, the ORF was inserted into the Rep41 expression vector with the nmt41 promoter. The *S. pombe* genomic maps were derived from ‘EnsemblFungi,’

(https://fungi.ensembl.org/Schizosaccharomyces_pombe/Info/Index). ‘+’ and ‘$-$’ indicate the suppression activity of the fragment.


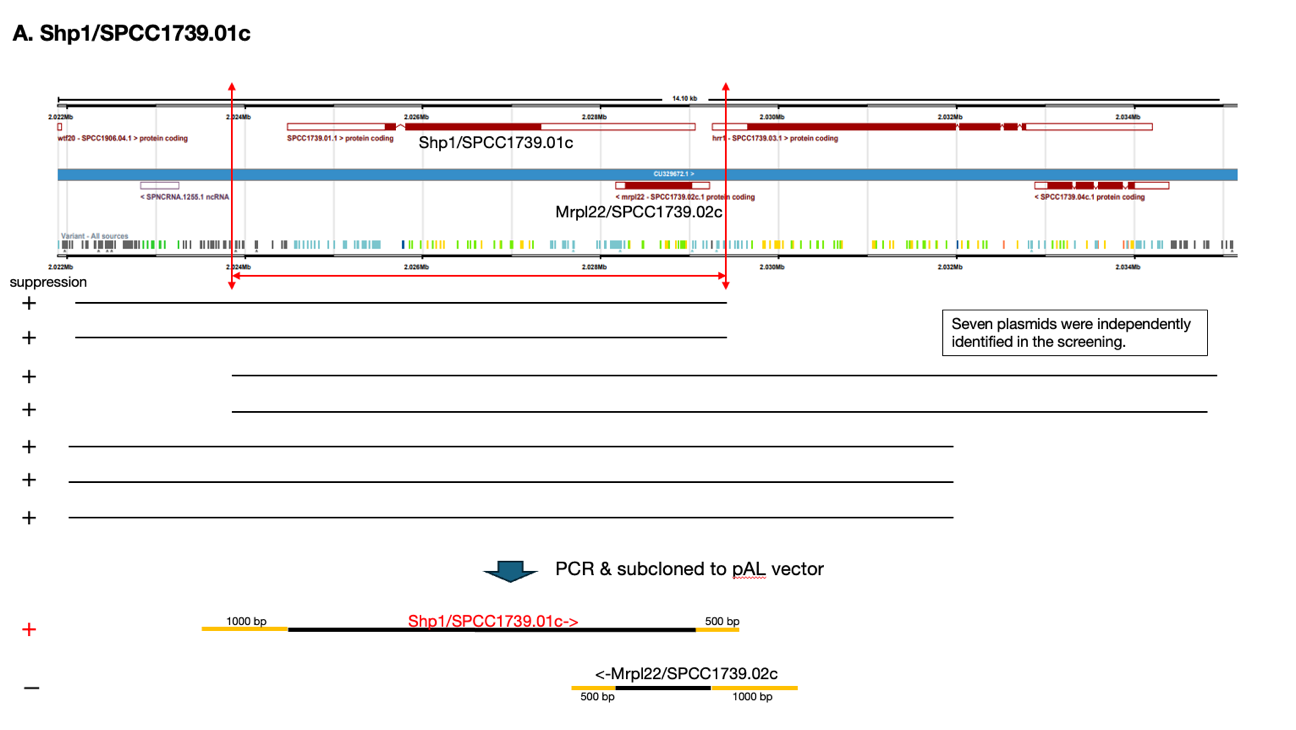


**Figure S1. The procedure of multicopy suppressor screening (continued)**


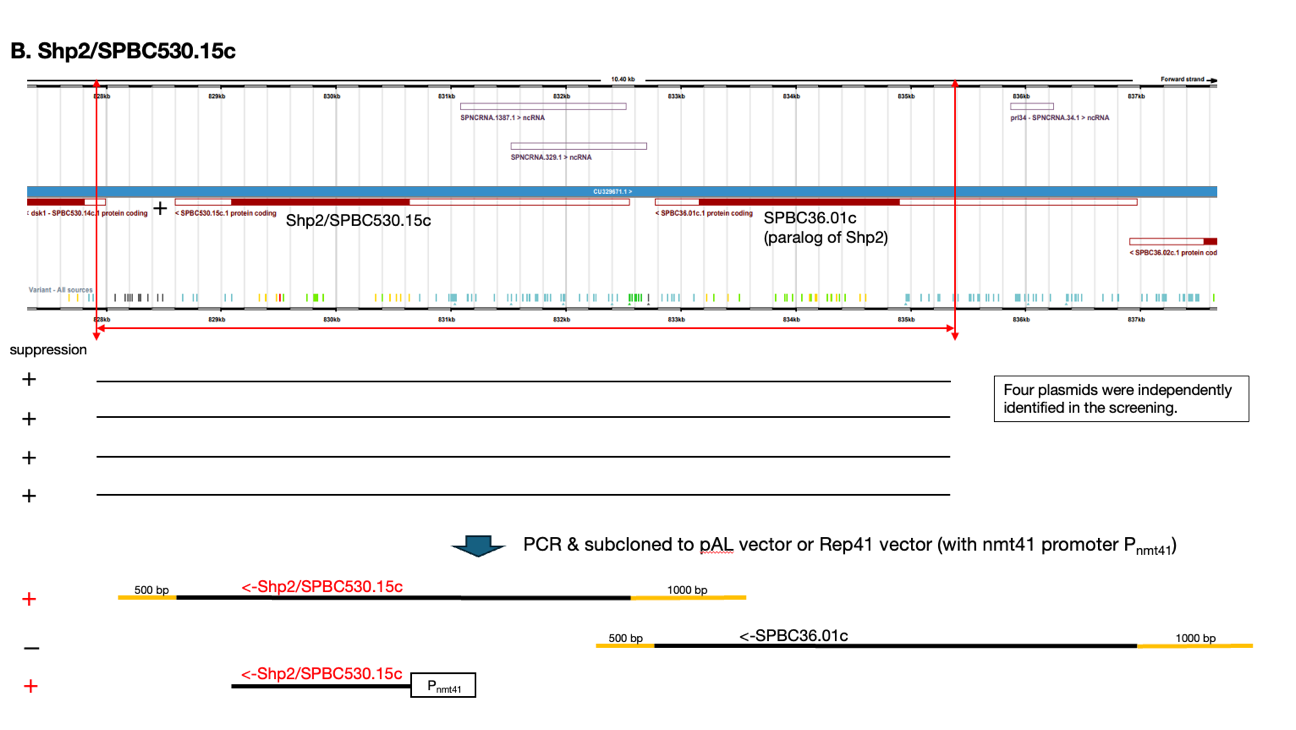


**
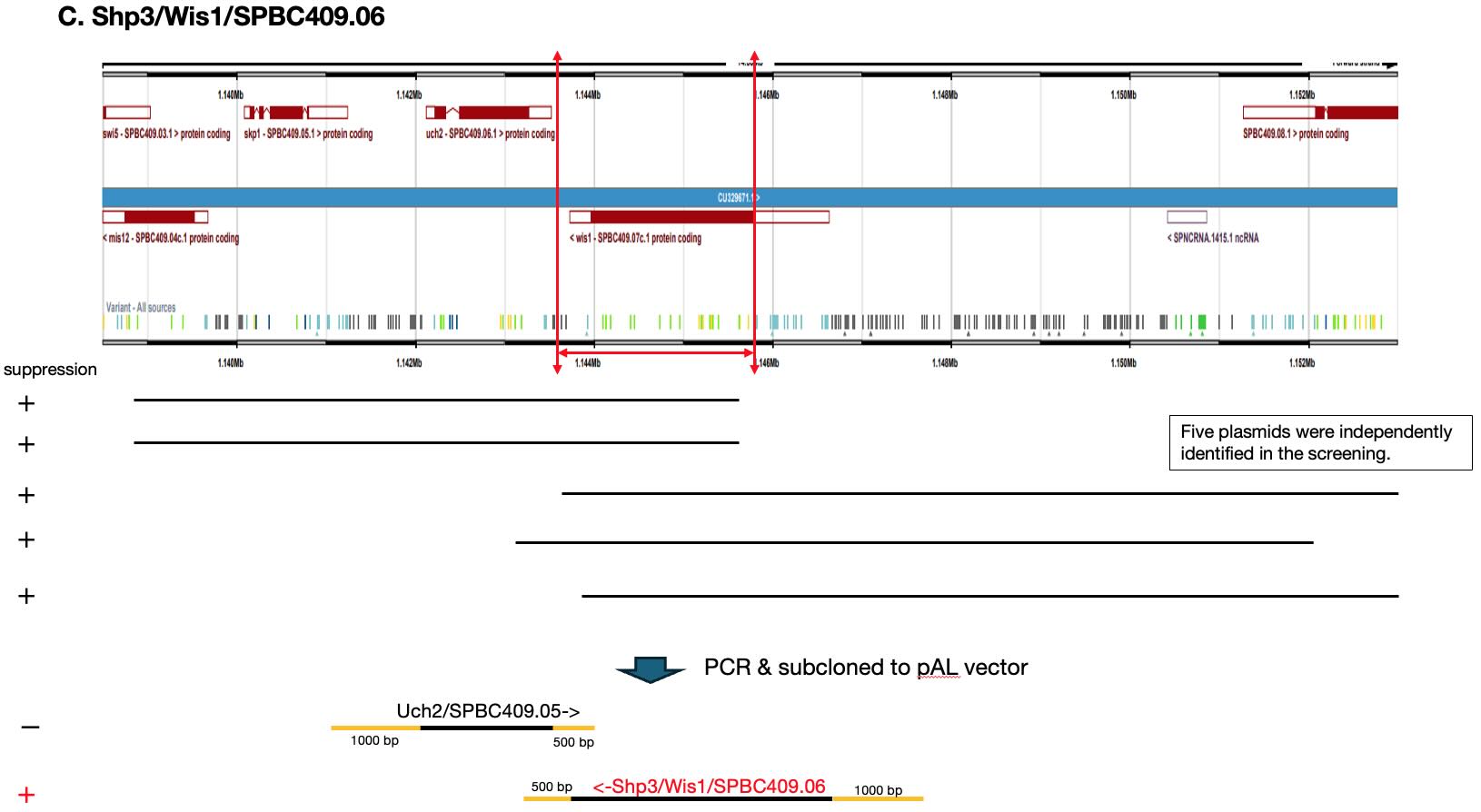
**


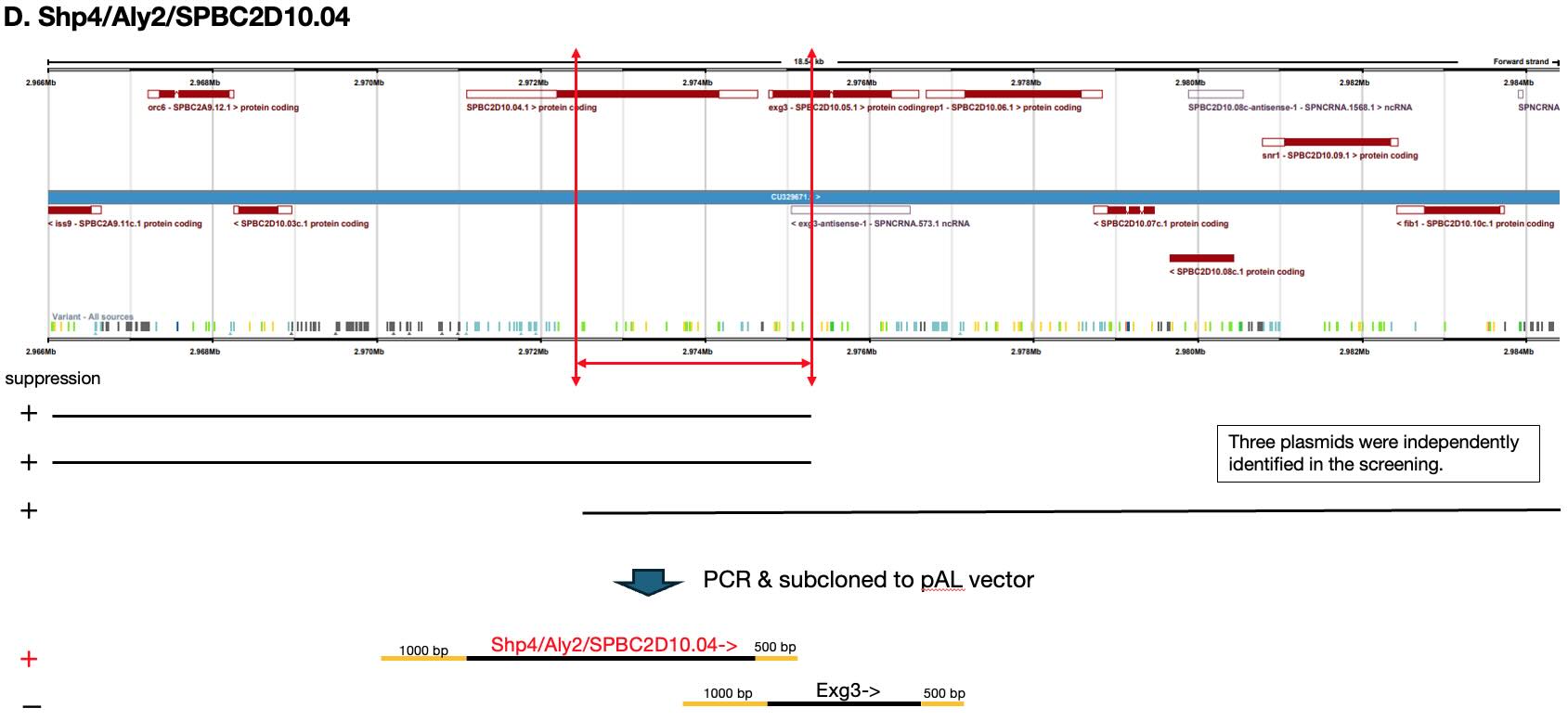
**Figure S1. The procedure of multicopy suppressor screening (continued)**


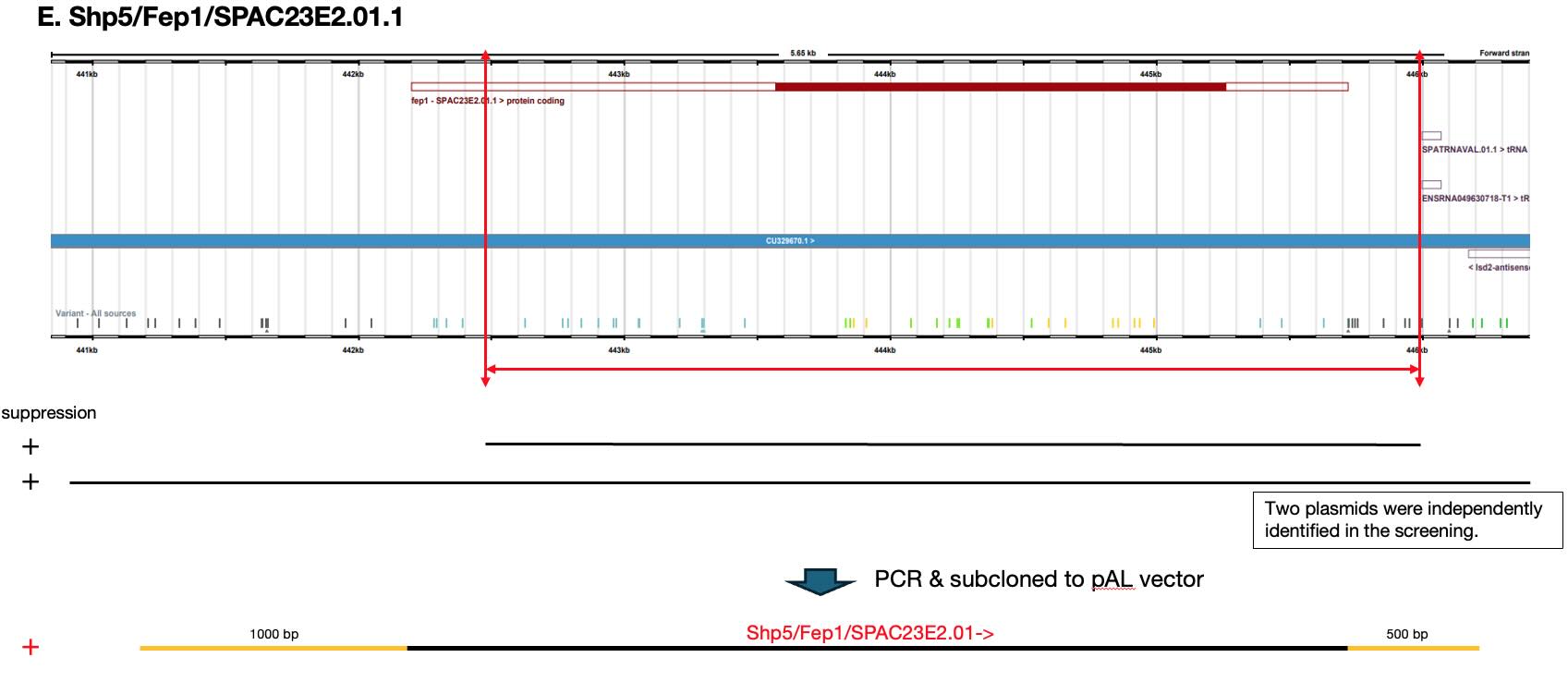


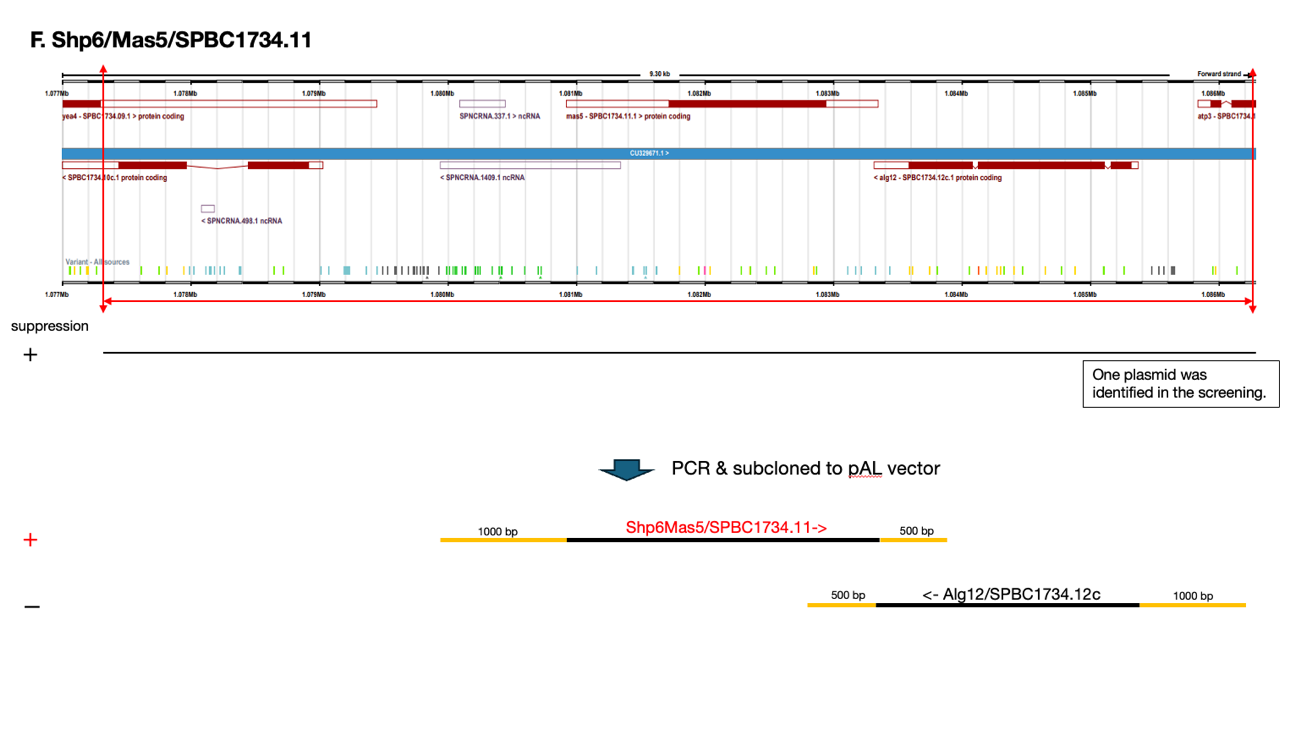
**Figure S1. The procedure of multicopy suppressor screening (continued)**


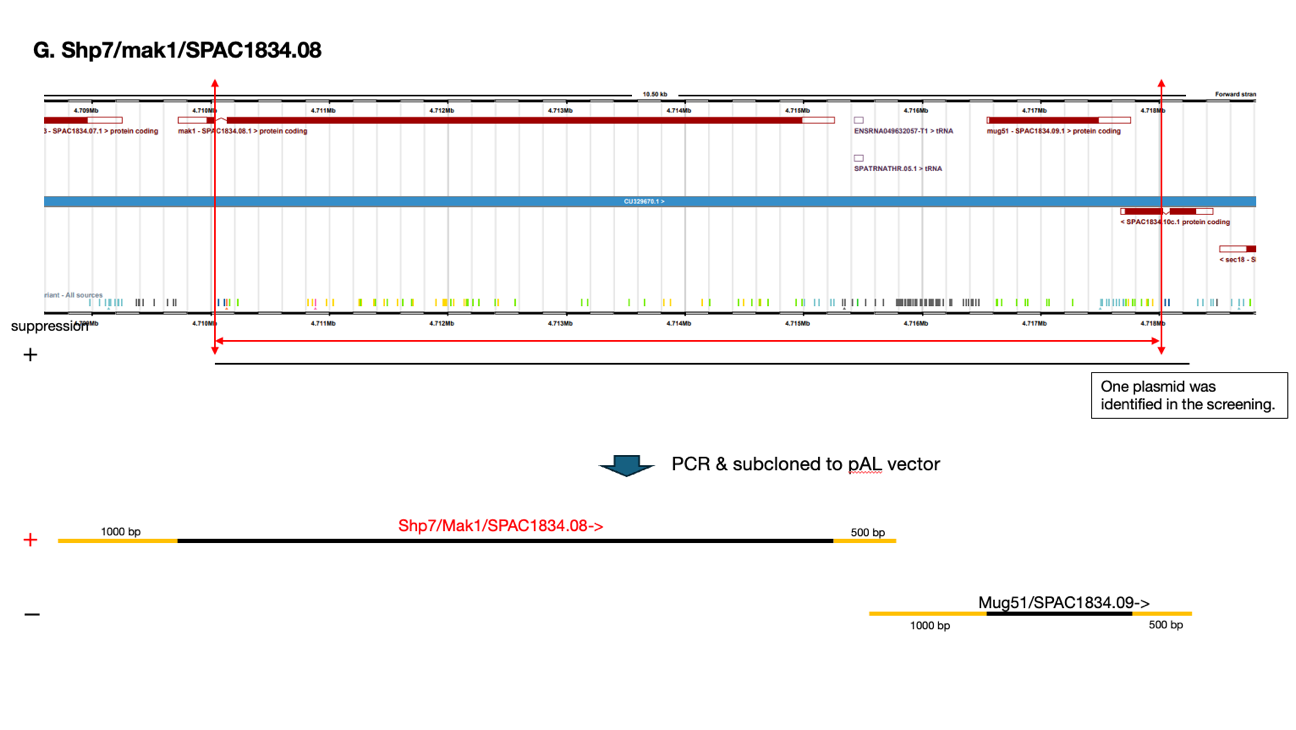


**Figure S1. The procedure of multicopy suppressor screening (continued)**

**
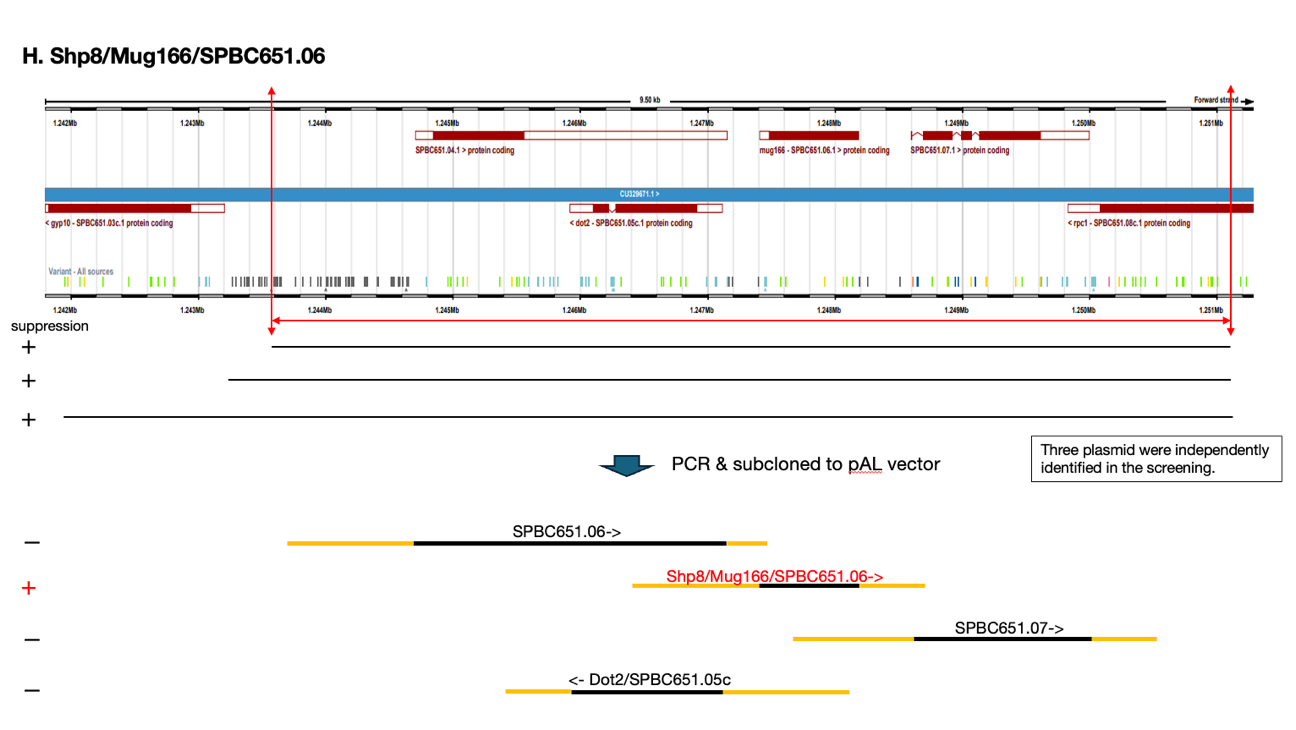
**

**
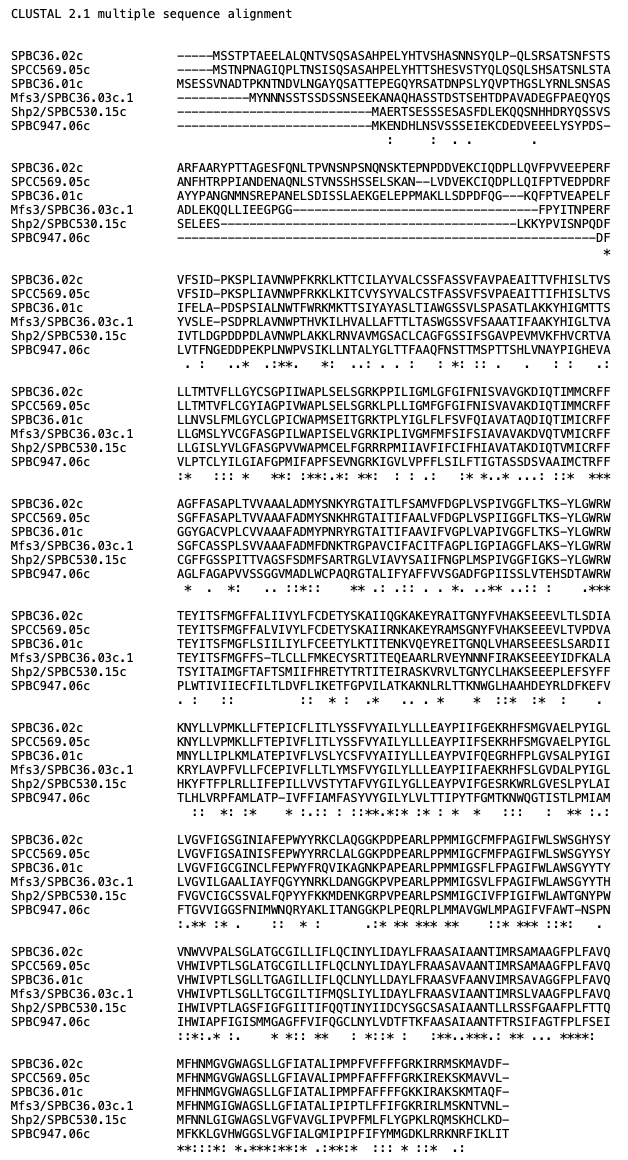
**

**Figure S2. The multiple alignments of the six putative polyamine/spermidine transporters in *S. pombe*.** The amino acid sequences of the six were analyzed with CLUSTAL 2.1 provided by GenomeNet (https://www.genome.jp). Alignment and phylogenetic reconstructions, shown in Fig. 3B, were performed using the function of ‘build’ of ETE3 3.1.2 as implemented on the GenomeNet [(https://www.genome.jp/tools/ete/)](https://www.genome.jp/tools/ete/).


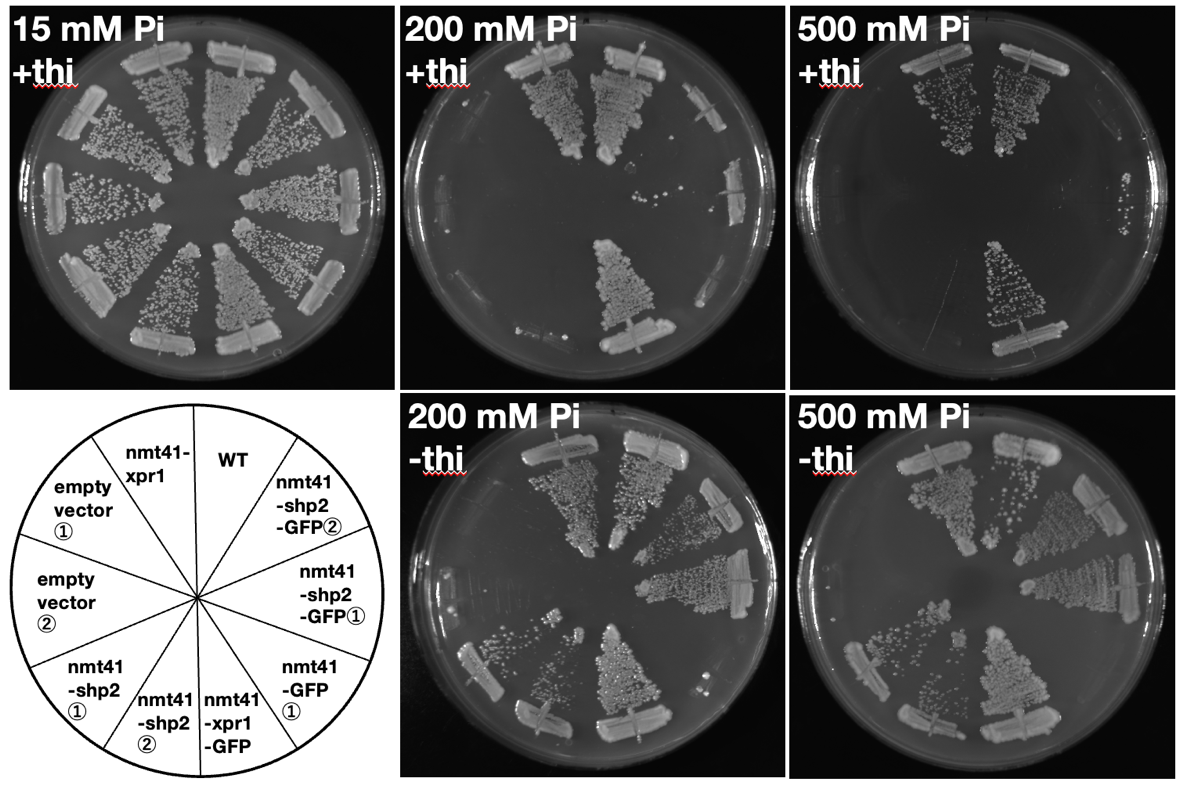


**Figure S3. Shp2-GFP suppresses the Pi hypersensitivity of *∆pqr1∆xpr1*.**

*∆pqr1∆xpr1* was transformed with plasmids expressing Shp2, Shp2-GFP, Xpr1, and Xpr1-GFP, driven by the *nmt41* promoter that is activated without thiamin (thi). WT indicates the indigenous WT 972. Induction of either Shp2 or Shp2-GFP rescued *∆pqr1∆xpr1* at higher Pi without thiamine (promoter ON). Either Xpr1 or Xpr1-GFP rescued *∆pqr1∆xpr1* even with thiamine (the promoter was suppressed); the leak-level transcription might be enough for the suppression. These agar plates were incubated at 30 °C for five days.

**
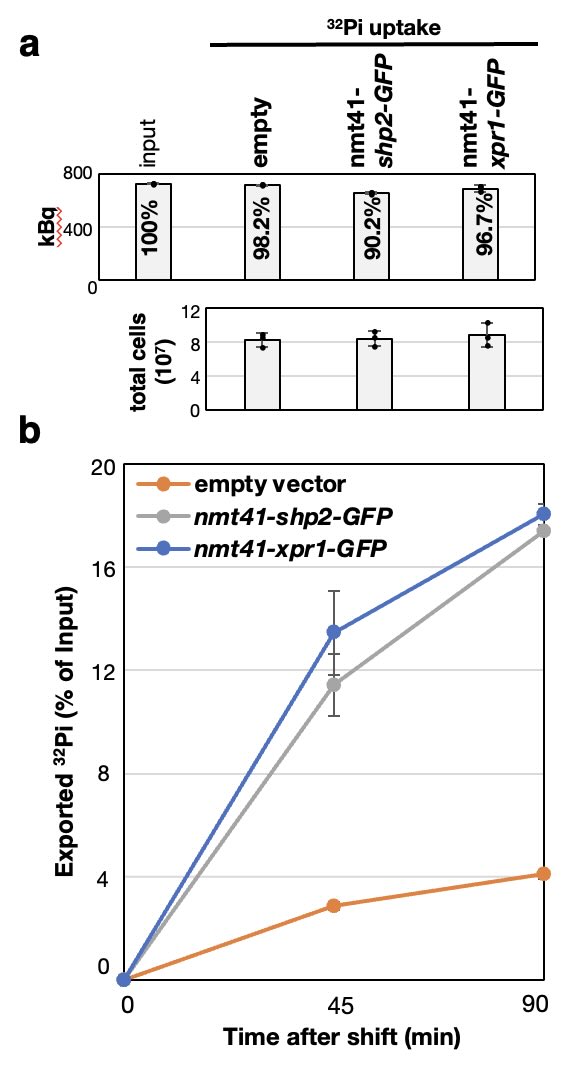
**

**Figure S4. The overproduction of Shp2 promotes the Pi export.**

***a*,** ^32^Pi uptake before the shift. After ^32^Pi incorporation, aliquots of cells of indicated strains were applied for a liquid scintillation counter. The amount of the input ^32^Pi was also measured and was compared with the incorporated ^32^Pi (upper). The percentages indicate ratios of ^32^Pi incorporated in the cells to the input. Cell numbers used in the assay are also shown (bottom). ***b***, The time-course analysis of ^32^Pi export in 15 mM Pi EMM2. The Y axis represents percentages of ^32^Pi exported from cells to total ^32^Pi incorporated. Experiments were repeated 3×. Individual data points, means, and SDs are presented.

**
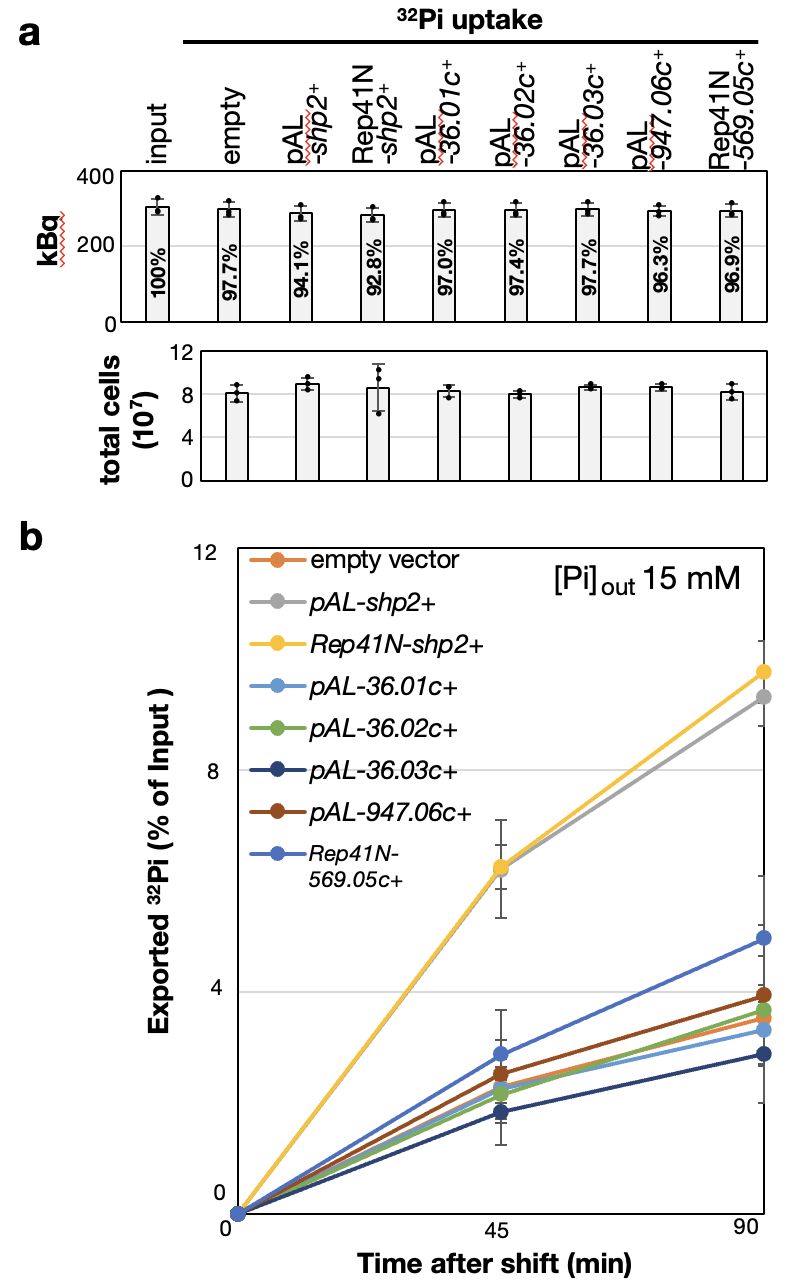
**

**Figure S5. Pi export assay with plasmids harboring the six putative spermidine transporters.**

***a***, ^32^Pi uptake before the shift. After ^32^Pi incorporation, aliquots of cells of indicated strains were applied for a liquid scintillation counter. The amount of the input ^32^Pi was also measured and was compared with the incorporated ^32^Pi (upper). The percentages indicate ratios of ^32^Pi incorporated in the cells to the input. Cell numbers used in the assay are also shown (bottom). As for the abbreviations, see the figure legends of Fig. 3. ***b***, The time-course analysis of ^32^Pi export in 15 mM Pi EMM2. The Y axis represents percentages of ^32^Pi exported from cells to total ^32^Pi incorporated. Experiments were repeated 3×. Individual data points, means, and SDs are presented.


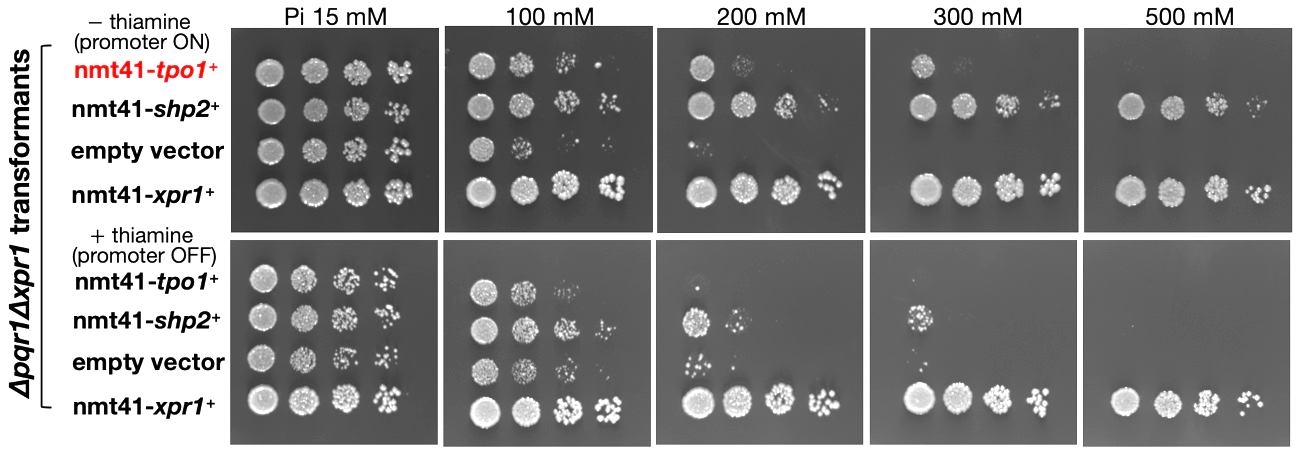


**Figure S6. Overproduction of Tpo1, *S. cerevisiae* SPD exporter, partially rescues *∆pqr1∆xpr1* as Shp2.**Tpo1, Shp2, and Xpr1 were overproduced by the *nmt41* promoter in *∆pqr1∆xpr1*. In the absence of thiamine (promoter ON, top), Tpo1-OP rescue the growth slightly but noticeably at 200 and 300 mM Pi. In the presence of thiamine (promoter OFF, bottom), such suppressions were not observed., suggesting that the growth recovery depends on Tpo1 expression. The experiments were replicated three times using transformants obtained independently and their results were virtually the same . The typical example was shown.


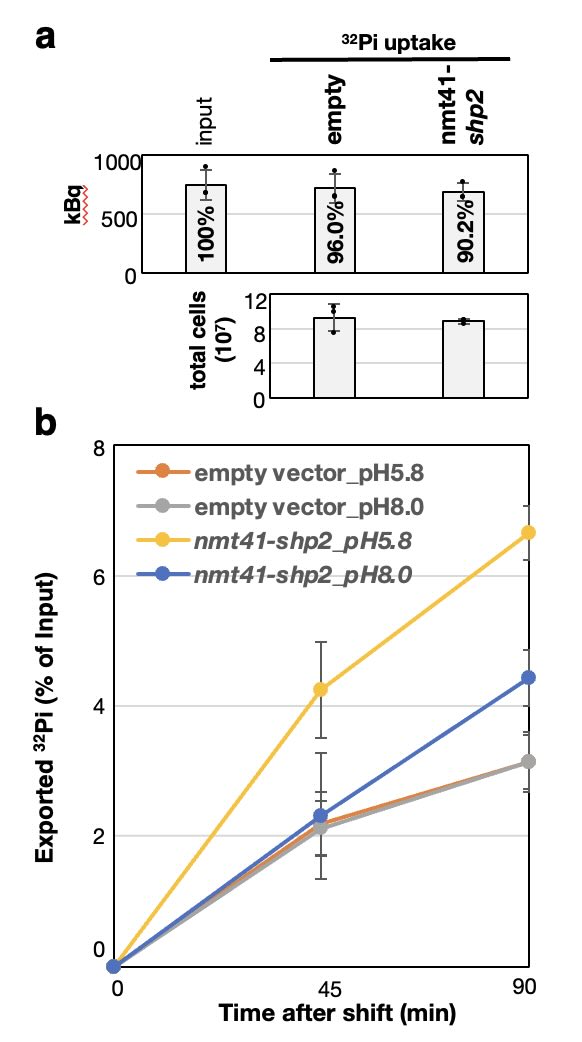


**Figure S7. Pi export assay at pH 5.8 and 8.0**

***a***, ^32^Pi uptake before the shift. After ^32^Pi incorporation, aliquots of cells of indicated strains were applied for a liquid scintillation counter. The amount of the input ^32^Pi was also measured and was compared with the incorporated ^32^Pi (upper). The percentages indicate ratios of ^32^Pi incorporated in the cells to the input. Cell numbers used in the assay are also shown (bottom). As for the abbreviations, see the figure legends of Fig. 3. ***b***, The time-course analysis of ^32^Pi export in the pH. 5.8 buffer and pH 8.0 buffer. The cells shown in a, which incorporated ^32^Pi, were split and suspended in the two buffers. The Y axis represents percentages of ^32^Pi exported from cells to total ^32^Pi incorporated. Experiments were repeated 3×. Individual data points, means, and SDs are presented.


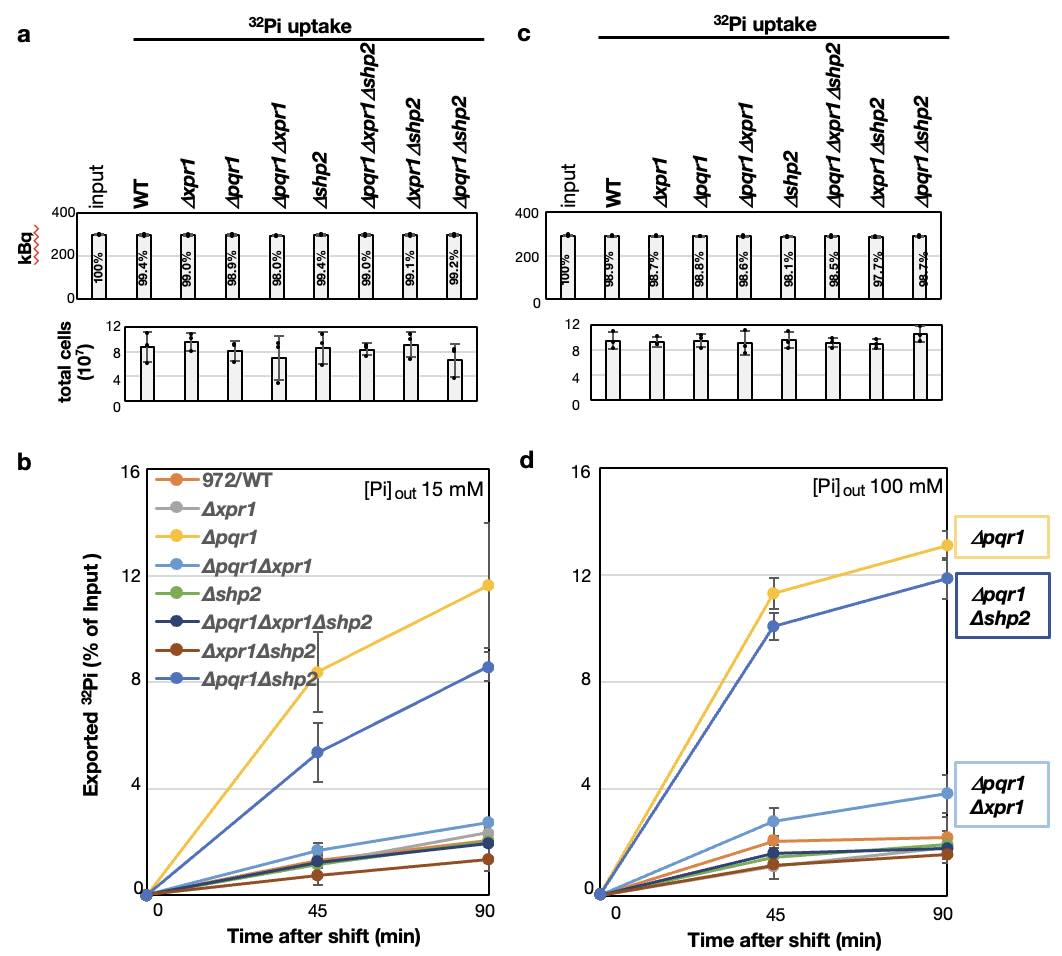


**Figure S8. Pi export assay with deletion mutants of *pqr1^+^*, *xpr1^+^*, *shp2^+^*, and their double and triple mutants.**
Pi export assays at [Pi] 15 mM (*a,b*) and 100 mM (*c,d*) are shown. ***a*** and ***c*** ^32^Pi uptake before the shift. After ^32^Pi incorporation, aliquots of cells of indicated strains were applied for a liquid scintillation counter. The amount of the input ^32^Pi was also measured and was compared with the incorporated ^32^Pi (upper). The percentages indicate ratios of ^32^Pi incorporated in the cells to the input. Cell numbers used in the assay are also shown (bottom). ***b*** and ***d***, the time-course analysis of ^32^Pi export in 15 mM Pi EMM2 (***b***) and 100 mM EMM2 (***d***). The Y axis represents percentages of ^32^Pi exported from cells to total ^32^Pi incorporated. Experiments were repeated 3×. Individual data points, means, and SDs are presented.
